# Tissue Specificity and Dynamics of Sex-Biased Gene Expression in a Common Frog Population with Differentiated, Yet Homomorphic, Sex Chromosomes

**DOI:** 10.3390/genes9060294

**Published:** 2018-06-12

**Authors:** Wen-Juan Ma, Paris Veltsos, Melissa A. Toups, Nicolas Rodrigues, Roberto Sermier, Daniel L. Jeffries, Nicolas Perrin

**Affiliations:** 1 Department of Ecology and Evolution, University of Lausanne, CH 1015 Lausanne, Switzerland; parisveltsos@gmail.com (P.V.); melissa.toups@ist.ac.at (M.A.T.); nicolas.rodrigues@unil.ch (N.R.); roberto.sermier@unil.ch (R.S.); DanielLee.Jeffries@unil.ch (D.L.J.); nicolas.perrin@unil.ch (N.P.); 2 Department of Biology, Indiana University, Jordan Hall, 1001 East Third Street, Bloomington, IN 47405, USA; 3 Institute of Science and Technology Austria, Am Campus 1, 3400 Klosterneuburg, Austria

**Keywords:** tissue specificity, gene expression, sex bias, development, adult tissues, rate of evolution, pleiotropy, sex chromosomes

## Abstract

Sex-biased genes are central to the study of sexual selection, sexual antagonism, and sex chromosome evolution. We describe a comprehensive de novo assembled transcriptome in the common frog *Rana temporaria* based on five developmental stages and three adult tissues from both sexes, obtained from a population with karyotypically homomorphic but genetically differentiated sex chromosomes. This allows the study of sex-biased gene expression throughout development, and its effect on the rate of gene evolution while accounting for pleiotropic expression, which is known to negatively correlate with the evolutionary rate. Overall, sex-biased genes had little overlap among developmental stages and adult tissues. Late developmental stages and gonad tissues had the highest numbers of stage- or tissue-specific genes. We find that pleiotropic gene expression is a better predictor than sex bias for the evolutionary rate of genes, though it often interacts with sex bias. Although genetically differentiated, the sex chromosomes were not enriched in sex-biased genes, possibly due to a very recent arrest of XY recombination. These results extend our understanding of the developmental dynamics, tissue specificity, and genomic localization of sex-biased genes.

## 1. Introduction

Sexual dimorphism is almost ubiquitous in sexual organisms. The genetic basis underlying sex differences often involves many genes and is very complex [1]. Males and females share the majority of their genome, which creates intralocus conflicts at many genes [2]. Differential gene expression between the sexes is an important mechanism for the resolution of intralocus conflicts and is responsible for the majority of sex-specific phenotypes [2,3]. Furthermore, differences in gene expression influence the variation in the degree of sexual dimorphism, as elegantly demonstrated by the transcriptomic comparison between subordinate and dominant male turkeys [4]. Sex-biased gene expression is widespread in animals [5,6,7,8,9,10] and is most evident in adults, in which sexual phenotypes are the most manifest [3,11,12].

Most studies of sex-biased gene expression have focused on model systems such as mammals, birds, and *Drosophila* [1,4,6,12,13,14,15], and have primarily used adult gonads, which show the highest degree of sex-biased gene expression among all organs [2,16]. A major conclusion from these studies has been that male-biased genes tend to be more numerous and to have a higher expression than female-biased or unbiased genes [13,17,18,19]. Other somatic tissues can exhibit highly sex-biased expression as well [2,12], but they have received less attention, even though they may be important for secondary sexual characters and behaviors, or indicate metabolic and other life-history differences between the sexes [20]. The dynamics of sex-biased gene expression throughout development have been the least studied [21], and previous studies detected little overlap in sex-biased genes throughout developmental stages and adults [1,3,22]. 

Sex-biased genes have an elevated rate of evolution compared to unbiased genes [7,23,24,25,26]. This is hypothesized to result from sex-specific selection (reviewed in [2]); however, different studies have found that either female- or male-biased genes evolve faster, depending on the species, or whether the tissue studied is embryonic or adult, at least in birds and *Drosophila*. Faster male-biased gene evolution has been documented in adult tissues of birds and *Drosophila* [7,13,25,27], and there is evidence that this rapid evolution is driven by positive selection [23,24,26]. However, studies on embryonic tissues in chicken, *Drosophila*, and mosquitos (both embryo and gonad tissues), as well as in fungi, have found elevated rates of evolution in female-biased genes [3,6,28,29,30,31], suggesting that other forces may influence the evolutionary rate of gene sequences, not just sexual selection, which is stronger in males. Given that the sex bias status of a gene is highly dynamic, a characterization of its consistency across developmental stages and different tissues is necessary before inferring the evolutionary forces affecting sex-biased genes [21]. 

Another force influencing the evolutionary rate of genes is their degree of pleiotropy. It has been hypothesized that genes with fewer and more specific functions would be more likely to respond to selection than genes with multiple functions. In the latter case, the response to selection that enhances one role of a multifunctional gene would be counteracted by the deterioration of other roles of the same gene [32,33]. The degree of pleiotropy of a gene has also been suggested to limit its ability to become sex biased [14,20]. The metric for tissue specificity, Tau (τ), combines the information of expression across tissues and has been used to describe the degree of pleiotropy of a gene [34,35]. Studies using τ have confirmed that narrowly expressed genes evolve faster than broadly expressed genes [14,20] and that sex-biased genes tend to have limited pleiotropy [12,36]. The relationships between the rate of gene evolution, tissue specificity, and sex bias are therefore complex, and may be different for sex-biased genes of different tissues.

A third evolutionary force affecting sequence evolution and sex-biased gene expression is sex linkage. Sex chromosomes are associated with asymmetric inheritance in the two sexes, which predicts that they should become enriched in sex-biased or sexually antagonistic genes [37,38,39]. Multiple studies have found that sex chromosome formation leads to major changes in gene expression, with X chromosomes becoming feminized and Z chromosomes masculinized (reviewed in [40]). These changes are evident in many species with highly degenerated sex chromosomes [10,41,42,43,44], as well as species with neo-sex chromosomes, such as threespine sticklebacks [9,45], and two *Drosophila* species, whose neo-sex chromosomes were generated by fusions between a degenerated Y and an autosome, 1–15 million years (Myr) ago [15,46]. However, the timing of the enrichment of sex-biased gene expression after the birth of a new sex chromosome is unclear, and the study of organisms at an early stage of sex-chromosome evolution is required.

The common frog, *Rana temporaria*, provides an ideal model to investigate the dynamics of sex-biased gene expression, tissue specificity, and sex linkage, as it possesses homomorphic sex chromosomes [47,48,49] and thus represents an early stage of sex-chromosome evolution. It is distributed widely throughout Europe [50] and has a variable sex-determination system ranging from genetic to non-genetic [47,51,52]. Interestingly, one population in Northern Sweden (Ammarnäs) has two fully differentiated sex chromosomes (chromosomes 1 and 2) [47,53], while one Southern Swedish population (Tvedöra) shows limited sex-chromosome differentiation for only one (chromosome 1), whose sex-specific region is only detected close to the candidate sex-determination gene *Dmrt1* (hence forming a proto-sex chromosome) [47,52]. Ammarnäs therefore potentially represents a more advanced stage of sex-chromosome evolution. In support, both sex chromosomes in Ammarnäs show elevated genetic differentiation (*F_st_*) and an enrichment for female-biased genes in gonad tissue [53], whereas our previous gene expression study on Tvedöra has revealed neither the enrichment of proto-sex chromosomes in sex-biased genes, nor a higher rate of evolution of sex-linked genes in juvenile stages [22]; however, an enrichment of female-biased gene expression on the proto-sex chromosome has been detected in gonad tissues [53]. One remaining question here is whether these signatures appear on more differentiated sex chromosomes across juvenile and adult tissues. 

In this study, we use RNA sequencing (RNAseq) data collected from five developmental stages and three adult tissues of both sexes from a *R. temporaria* population in Ammarnäs, to investigate the interaction between tissue specificity and sex-biased gene expression in the context of their influence on the gene evolutionary rate. We first characterize the tissue specificity of genes expressed in different tissues, then describe sex-biased gene expression across development to adulthood and their effects on coding sequence evolution, and finally investigate their genomic locations and discuss the findings in the context of homomorphic but fully differentiated sex chromosomes. 

## 2. Materials and Methods

### 2.1. Field Sampling and Rearing Conditions

Twelve mating pairs in amplexus were caught during the 2015 breeding season in Northern Sweden near Ammarnäs (65°54′ N/16°18′ E), a population previously studied for sex-chromosome differentiation [47,52]. All adults were sampled for buccal cells with sterile cotton swabs, and mating pairs were individually kept overnight in 11 L plastic boxes with grass tufts and half-filled with pond water, allowing them to lay a clutch. Following this, 24–36 h post-mating, nine females and seven males were anaesthetized and euthanized in 0.2% ethyl3-aminobenzoate methanesulfonate salt solution (MS222), and the gonads, liver, and brain tissues were removed before severing the brain stem. Five samples of each tissue were stored in RNAlater (Qiagen, AMBION, Inc., Austin, TX, USA) and transported to the University of Lausanne. The remaining eight adults were immediately released at the place of capture. 

Five clutches were brought back to the University of Lausanne, where they were reared in separate tanks in a climatic room at constant conditions (15~16 °C with 12:12 light:dark cycle). Juveniles were first fed fish-flakes, then fruit flies and small crickets after metamorphosis. One to four offspring from each clutch were sampled at each of the five developmental stages (Gosner stage, G): G23, G27, G31, G43 (metamorph; 1.1–1.4 cm snout-vent length), and G46 (froglet; 1.8–2.2 cm snout-vent length) [54]. At our rearing conditions, sampling took place 20 days, 29 days, 85 days, seven-eight months, and ten months after spawning, respectively. These stages represent important points regarding sex determination and differentiation. Gonadal development is first initiated at stage G27, with histological differentiation visible from stage G31, morphological gonad differentiation visible under microscopy from stage G43 (metamorphosis), and gonad differentiation is completed at stage G46 (froglet). Sampled juveniles were anaesthetized and euthanized in MS222, then immediately plunged in RNAlater (Qiagen). The tail tip from each tadpole, and a toe clip from metamorphs and froglets, were cut for genotyping. Samples of the two latter stages (G43 and G46) were dissected for phenotypic sex determination (see below), and their digestive tracts (stomach, small intestine, large intestine) were removed to limit the contamination of RNA analyses by food remains and microorganisms. Samples in RNAlater were preserved at −20 °C up to 10 months before RNA extraction. 

Ethical permits were provided by the Swedish Board of Agriculture (C 6/15) and by the Veterinary Office of the Vaud Canton, Switzerland (authorization 2287).

### 2.2. Genotyping

As phenotypic sex cannot be assessed prior to stage G43, we genotyped all sampled individuals to assess their genotypic sex using three markers with Y-diagnostic alleles (namely *Dmrt1*-1, *Dmrt1*-2, *Dmrt1*-5) (primer sequences from [52]; Supplementary Table S1). After an overnight treatment at 56 °C with tissue lysis buffer ATL and 20% proteinase K (Qiagen), PCR reactions were performed in a total volume of 10 µL, including 3 µL of extracted DNA, 2.22 µL of Milli-Q water, 3 µL of Qiagen Multiplex Master Mix, 0.14 to 0.3 µL of labeled forward primer, and 0.14 to 0.3 µL of unlabeled reverse primer (in total 1.78 µL of primer mix). PCRs were conducted on Perkin Elmer 2700 machines using the following thermal profile: 15 min of Hot Start Taq polymerase activation at 95 °C, followed by 35 cycles including denaturation at 94 °C for 30 s, annealing at 55 °C for 1.5 min, and elongation at 72 °C for 1 min, ending the PCR with a final elongation of 30 min at 60 °C. PCR products were then analyzed on an automated ABI Prism 3100 sequencer (Applied Biosystems, Foster City, CA, USA) and alleles were scored using GENEMAPPER v. 4.0 (Applied Biosystems).

### 2.3. Phenotypic Sex

The phenotypic sex of G43 and G46 samples was determined based on gonad morphology, following dissection in RNAlater (Qiagen) under a binocular microscope. Ovaries in common frogs develop from the whole gonadal primordia into a large whitish/yellowish structure with distinct lobes and a characteristic granular aspect conferred by the many oocytes embedded in the cortex [55,56]. In contrast, testes develop from the anterior part of the gonadal primordia only (the posterior part degenerates) into a small oblong structure, with a smooth cortex covered with melanic spots [55,56]. Each individual was scored as phenotypically male, phenotypically female, or undifferentiated, following the gonad-scoring description in [52]. 

### 2.4. RNA Extraction and Sequencing 

For adult tissues, the RNA extraction, RNA-seq library preparation, and RNA sequencing on the NextSeq500 platform were performed with standard Illumina protocols by the sequencing company Microsynth (Balgach, Switzerland). The sequenced RNA yielded a total of 675.7 million (75 bp paired-end) reads for adult tissues. Data for gonadal tissue was previously analyzed in [53]. 

For developmental stages, in order to maximize the independence of biological replicates, we selected, for each stage, at least one female (XX) and one male (XY) individual from each of three clutches. Additionally, we also selected a few individuals from two more clutches due to occasional low replicates at stages G43 and G46, based on the genotyping results (Supplementary Table S1). This equated to a total of 32 RNA samples across five developmental stages. RNA was extracted from whole bodies for the earliest three stages, because individuals are too small to reliably extract RNA from particular tissues. For the later stages G43 and G46, whole bodies were also used in order to have comparable datasets with the earliest three stages. RNAseq analyses are thus expected to capture allometric differences of organs between stages. RNA extractions were performed following a mixed Trizol/Qiagen columns protocol. We followed the normal Trizol protocol until the two phase stage (apolar and aqueous phase). We took 500 µL of the aqueous phase, added 300 µL of ethanol, and loaded the mix in an RNeasy column (Qiagen), and then followed the standard Qiagen RNeasy protocol. Each RNA-later preserved sample was individually homogenized in Trizol (Life Technologies, Carlsbad, CA, USA), followed by phase separation (using chloroform). After ethanol precipitation of the upper phase, RNA was washed with 70% ethanol twice and collected, followed by a DNase digestion step. RNA libraries were then prepared and barcoded at the Lausanne Genomic Technologies Facility, University of Lausanne using standard protocols. Six RNA libraries were multiplexed per lane, and were sequenced on an Illumina HiSeq 2500 (Illumina Inc., San Diego, CA, USA), resulting in a total of 2.69 billion (100 bp paired-end) reads for five developmental stages. 

### 2.5. De Novo Transcriptome Assembly and Assigning Transcripts to Chromosomes

Raw RNAseq reads were trimmed before the assembly. We assessed the sequencing data quality using FastQC v10.5 (Babraham Bioinformatics, Babraham Institute, Cambridge, UK). Trimmomatic 0.36 [57] was used to filter reads containing adapters and to trim reads if the leading and trailing bases with a Phred score <4. We also trimmed sequences immediately after the four base average Phred quality score dropped below 15, and excluded read pairs if either read was shorter than 36 bp after trimming. After filtering, there was an average of 23.3 million reads per adult tissue sample, and for tadpoles and froglets, there was an average of 84.2 million reads per sample. In order to capture gene expression in both developmental stages and adult tissues, we randomly selected one individual from each sex of the three adult tissues (gonad, brain, liver), and one sample from each of the five developmental stages (G23, G27, G31, G43, G46) for our transcriptome assembly. 

We built a de novo transcriptome using Trinity v.2.3.0 with default parameters [58]. We filtered the assembly for transcripts with a minimum length of 300 bp. These were then filtered transcripts with possible transposable element insertions by masking the transcriptome assembly using a custom repeat library for *R. temporaria* using RepeatMasker [59] (Supplementary Text S1), only retaining transcripts that were at least 75% unmasked. Pseudoalignments were generated for the tissues and stages used in the initial assembly using Kallisto v.0.43.0 and transcripts with a minimum of 1 transcripts per million (TPM) in the male tissues, female tissues, or tadpole/froglet tissues were retained. We then selected the highest expressed transcript per gene model as defined by Trinity for the final transcriptome, which contained 44,635 transcripts.

As synteny is extremely well preserved across anurans [60,61,62], we could map our transcripts to the genome of the African clawed frog, *Xenopus tropicalis*, the most closely related anuran with a high quality reference [63]. Open reading frames (ORFs) were extracted from the transcripts using TransDecoder-3.0.1 [58]. We reciprocally blasted these ORFs to peptide sequences from *X. tropicalis*. One-to-one orthologs were identified when the single best match between the transcript and peptide had an e-value less than 10^−10^, an overlap of at least 50% of both the transcript and peptide, and a minimum identity of 40%. These criteria assigned 11,383 transcripts in *R. temporaria* to the genomic positions of their orthologs in *X. tropicalis*, with no significant bias among chromosomes (except for a slight deficit on chromosomes 7–9; Supplementary Table S2). 

### 2.6. Tissue Specificity and Tissue-Specific Gene Expression

Histological differentiation of gonads is reported to start at stage G31 in *R. temporaria* [55,56]. No sex differences are therefore expected in larval tissues prior to stage G31, so we combined both sexes for calculation of the tissue/stage specificity index. Gene expression measurements were analyzed from 11 stages/tissues, including five sex-pooled (G23, G27, G31, brain, liver), and six sex-limited (female G43, male G43, female G46, male G46, testis, and ovary). From these data, we then calculated tissue specificity, Tau (τ), for each expressed gene [34] with the formula:

(1) $$\tau =\frac{\Sigma_{i}\left[1-Log\left({TPM}_{i}\right)/Log\left({TPM}_{max}\right)\right]}{N-1},$$

where *N* is the number of stages/tissues examined and *TPM_max_* is the highest expression level detected for a given gene over all stages or tissues examined. Data for each gene was standardized to the number of TPM. *TPM_i_* is a measure of the number of transcripts detected for a given gene per million transcripts analyzed from tissue *i*; this standardization effectively corrects for gene leakage, where genes that do not necessarily function in a tissue are nonetheless transcribed at very low levels within that tissue or are passively transported to that tissue from other parts of the body [20]. The value of τ theoretically ranges from 0 to 1, with lower values indicating an expression pattern that is evenly distributed through all tissues examined, and higher values indicating more variation in expression levels across tissues and a greater degree of tissue specificity. Following the suggestion from a benchmark paper on tissue specificity [35], we considered genes with τ > 0.8 as tissue-specific genes, and then calculated the number of tissue-specific genes among developmental stages and adult tissues. 

To obtain an overview of gene expression patterns among all samples (adult, embryonic tissues with both sexes), we performed a principal component analysis (PCA). The analyses were conducted on normalized raw count data using EdgeR v3.4 [64] (see below section for details), using the “prcomp” function from the ggfortify library version 0.4.4 [65] and visualization of PCA plots with ggplot2 libraries version 2.2.1 in R version 3.4.3.

To investigate the relationship between sex-biased gene expression (measured as log2(male/female)) and tissue specificity, we performed Spearman’s rank correlation tests for each tissue with at least 100 sex-biased genes. Furthermore, we quantified the relative roles of sex bias and tissue specificity on the rate of coding sequence evolution, ratio of non-synonymous to synonymous substitutions (*dN*/*dS*), using linear models (after square root transform of *dN*/*dS* ratios), with sex bias, tissue specificity, and their interaction as explanatory variables. All statistical analyses were performed in R version 3.4.3.

### 2.7. Sex-Biased Gene Expression Analysis

To quantify gene expression, we mapped the trimmed reads of all 32 embryonic samples and 29 adult tissues to the filtered assembled transcriptome with Kallisto v.0.43.0 [66]. Read counts of the output from Kallisto mapping were imported for gene expression analysis in EdgeR v3.4 [64]. We filtered the low counts and kept genes with average Log(CPM) > 0 per sample, and CPM > 1 in at least half of the samples for each genetic sex per developmental stage or adult tissue. We then normalized the expression using the weighted trimmed mean of M-values (TMM) implemented in EdgeR, which is a scaling factor for library sizes that minimizes the log-fold change between samples [67]. We explored the libraries per stage in two dimensions using multi-dimensional scaling (MDS) plots (Supplementary Figure S1A–H). Normalized expression counts for each sample were used to calculate sex bias using standard measures. We first identified sex-biased genes based on the overall expression of each comparison group, and using Benjamini-Hochberg correction for multiple testing with a false discovery rate (FDR) of 5%. We identified sex-biased genes for each developmental stage separately. Sex bias was classified into four categories of fold changes, namely 2 (low), 2–4 (mild), 4–8 (high), and >8 (very high), and expressed as the log_2_ ratio of male-to-female expression (which has negative values for female-biased genes and positive values for male-biased genes). As suggested by [68], only fold changes (FC) ≥ 2 were considered to be sex-biased, in order to minimize possible scaling issues due to whole-body sampling (ovaries are slightly larger than testes, which may potentially lead to bias in calling sex-biased gene expression in embryos). Thus, unless stated otherwise, both conditions FDR < 0.05 and |log_2_FC| ≥ 1 will have to be met when calling sex bias. 

To investigate whether increased sex-biased gene expression was due to expression changes in male or female tissues, we compared normalized read counts (Reads Per Kilobase Million (Log_2_RPKM) obtained from edgeR v3.4 [64]) of sex-biased transcripts defined at various log_2_FC thresholds (≥1, ≥3, ≥5, ≥7) from males and females. Sex-specific genes were included in these analyses and a value ten times smaller than the minimum RPKM of any gene was added to the genes with zero counts, i.e., similar to [17], we consider sex-specific genes as extreme sex-biased genes. This allows us to analyze genes with a very high expression in one sex. These analyses were conducted in tissues with at least 100 sex-biased genes (G43, G46, brain, gonad, and liver). 

### 2.8. Tests for Enrichment of Sex Chromosomes in Sex-Biased Expression

We tested for the enrichment of sex chromosomes in sex-biased expression for each tissue and stage using two approaches. First, we assessed whether sex chromosomes had a higher proportion of sex-biased genes than autosomes using permutation tests. For each chromosome, we determined the number of transcripts assigned to that chromosome. Then, we randomly sampled that number from all assigned transcripts 10^4^ times to construct a null distribution. We assigned *p*-values based on the actual number of transcripts that were male-biased or female-biased relative to our null distribution. *p*-values were adjusted for multiple testing using a Bonferroni correction. In our second test, we directly compared the log_2_ ratio of male to female expression between sex chromosomes and autosomes, and assessed the significance using Wilcoxon tests. To characterize the pattern along the sex chromosomes (1 and 2), moving averages of gene expression ratios and sequence divergence were calculated in R v3.4.0 [69] with window size 40, based on sliding window analysis using the Rollmean and Rollapply functions in the Zoo package version 1.8-1 in R [70]. 

### 2.9. Faster-XY Evolution 

Faster X effects have been examined previously in *R. temporaria* [22,53]. To assess possible fast evolution of the Y allele compared to the autosomes (fast-Y effects), we used a transcriptome which we assembled de novo from the brain, liver, and testes of a single male to maximize the contribution of the Y alleles in the gene evolutionary rate calculation. Our power to detect the Y contribution is diluted by the inclusion of the X sequence. However, since there is no evidence of Faster-X evolution [22,53], any increase in the sequence rate on the sex chromosome would be attributable to the Y. The method of de novo transcriptome assembly and assignment of ortholog is the same as previously described. *dN*/*dS* was calculated between transcripts and *X. tropicalis* orthologs using the yn00 model in PAML v.4.9e [71]. We compared the *dN*/*dS* ratio of orthologs from the sex chromosomes (chromosomes 1 and 2) and autosomes. All statistics were performed in R v3.4.0 unless specified otherwise [69].

## 3. Results

### 3.1. De Novo Transcriptome Assembly 

We used RNA from five developmental stages and three adult tissues from both sexes to construct a de novo transcriptome assembly. De novo transcriptome assemblies typically consist of more contigs than can possibly be considered real transcripts, even when alternative splicing is taken into account [13]. After filtering to exclude transcripts with low expression or that had high similarity to other transcripts, we obtained a reference transcriptome containing 44,635 transcripts, which was used in expression analysis. BUSCO v2.0.1 [72] identified >85% complete and 5.2% fragmented single-copy tetrapod orthologs (*n* = 3950, C:85.8% [S:80.2%, D:5.6%], F:5.2%, M:9.0%). For simplicity, we refer to female-biased and male-biased for the gene expression comparison between XX and XY individuals, even though at early stages, only genotypic sex could be identified.

### 3.2. Gene Expression and Tissue-Specific Expression among Eight Stages/Tissues

**Figure 1:**
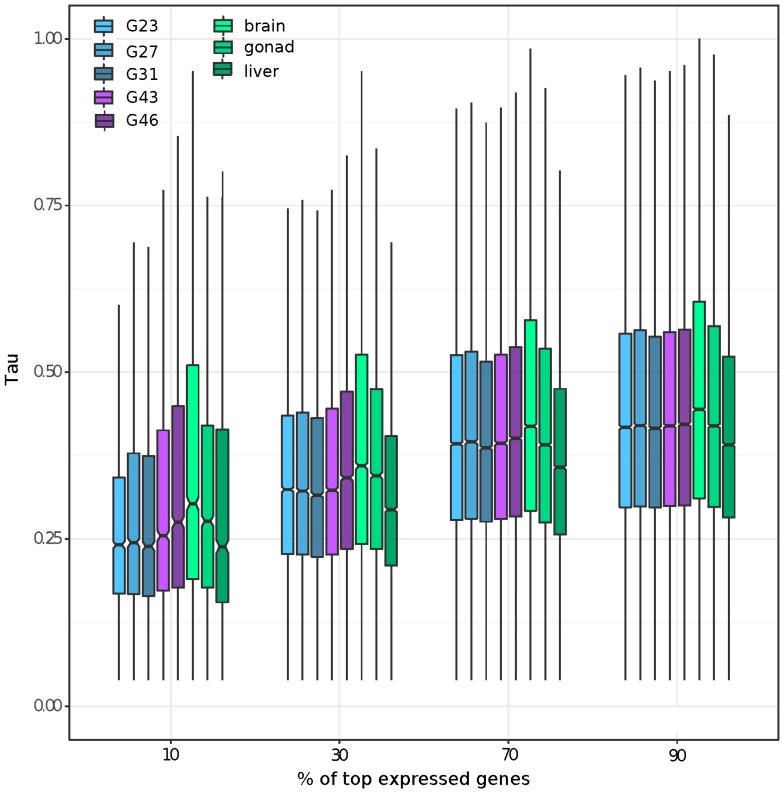
Summary of tissue specificity of the most expressed genes at different expression thresholds (top 10%, 30%, 70%, and 90%) of all eight studied tissues/stages.

The first two PCA components of the normalized count data explained ~41% of variance in gene expression among the developmental stages and adult tissues (Supplementary Figure S2a). PC3 (~15.53% of variance) distinguished the sexes for gonads and G46, and PC4 (~8.26% of variance) distinguished the sexes only for gonads (Supplementary Figure S2b). Components 5 and 6 (5.07% and 4.27% of variance, respectively) primarily distinguished testis tissue from all other samples (Supplementary Figure S2c). We further characterized the tissue specificity of genes expressed at different magnitudes within each tissue by plotting the Tau of the top 90, 70, 30, and 10% most expressed genes in each tissue. We found that genes expressed in brains had the highest tissue specificity, regardless of their expression level. Furthermore, genes with high expression (top 10% and 30%) showed a higher tissue specificity for gonad and developmental stages including reproductive organs (e.g., G43, G46), than somatic and early embryonic tissues (Figure 1). By defining genes as tissue or embryonic-stage specific as those with Tau > 0.8, we identified the brain to have the highest number of tissue-specific genes (2301), followed by gonad (1331), G46 (1142), and G43 (1078); each of the remaining samples had less than 800 tissue or embryonic-stage specific genes. The pattern remained the same if we restricted the definition with Tau > 0.9, with a lower number of tissue specific genes for each tissue/stage (brain: 771, followed by gonad: 598, G46: 294 and G43: 173; and each of the remaining tissue with less than 105).

### 3.3. Dynamics of Sex-Biased Gene Expression across Stages/Tissues and Chromosomes

**Figure 2:**
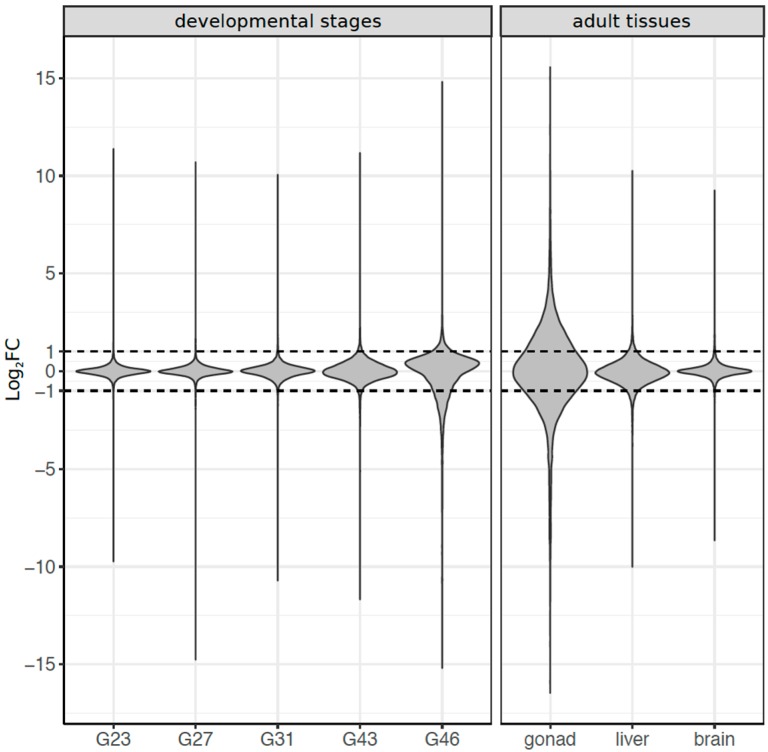
Summary of sex-biased gene expression Log_2_FC (fold change) in all eight tissues/stages. The dashed line defines the Log_2_FC threshold beyond which a gene was considered sex biased, with >1 indicating male bias and <−1 for female bias.

Among developmental stages, sex bias in gene expression increased with development and had the peak expression at stage G46 (froglet; Figure 2). Overall, 4924 transcripts (11% of total) were significantly sex-biased in expression in at least one of the five developmental stages (FDR < 0.05, |log_2_FC| ≥ 1). At early stages (G23 to G31), only a few genes were sex biased, with a marginally significant excess of male-biased genes at G23, but not in G27, nor G31 (Table 1). Sex bias increased at the metamorph stage G43 (186 genes) and dramatically so at the froglet stage G46 (4678 genes). Both metamorphs and froglets had a higher absolute expression of female-biased than male-biased genes, regardless of the fold-change threshold used to define sex bias (Figure 2, Table 1). No genes were consistently sex biased across development (Supplementary Figure S3a), few were shared between adjacent developmental stages, and most of the overlap did not differ from random expectation (Supplementary Table S3), suggesting a rapid sex bias turnover among stages. 

In adult tissues, 11,301 transcripts (25%) showed sex bias in at least one of the three tissues (FDR < 5%; |log_2_FC| ≥ 1), and gonads had the highest number of sex-biased genes (Figure 2, Table 1). There was limited overlap for sex bias among the three adult tissues (~2%, Supplementary Figure S3b), and the majority (96.6%) of sex-biased transcripts of adult tissues were identified in gonads, where there was a significant excess of male-biased genes (5,687 vs. 5,227 female-biased genes, Chi-Square test, *p* < 0.001). The other somatic tissues (brain and liver) had an excess of female-biased genes (Table 1, Supplementary Figure S4). 

**Figure 3:**
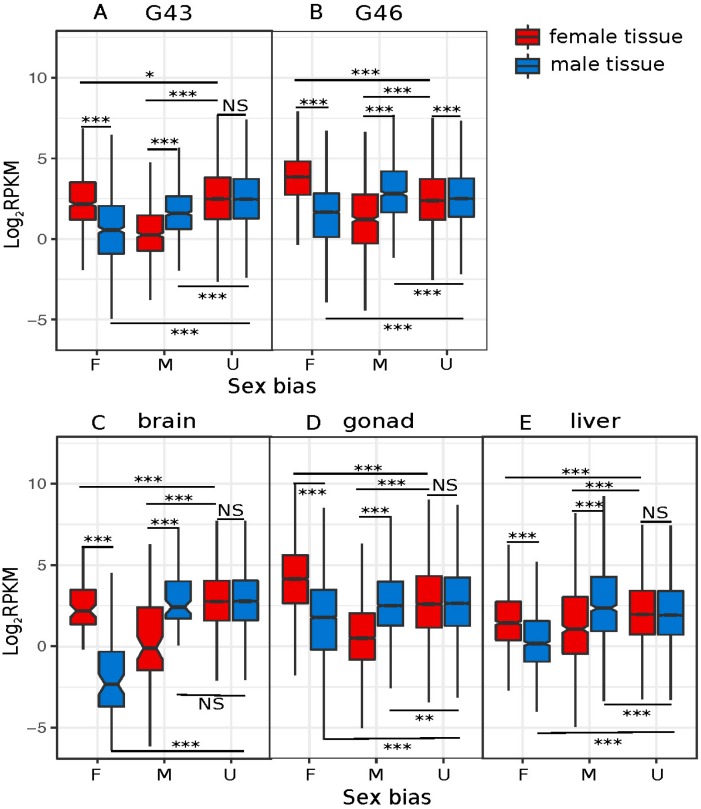
Breakdown of reads per kilobase million (RPKM) by sex bias and sample sex, for the five tissues/stages with >100 sex-biased genes. A: G43, B: G46, C: brain, D: gonad, and E: liver. *p* values of Wilcoxon rank sum tests are summarized above and below the box plots (NS = non-significant, *** = *p* < 0.001, ** = *p* < 0.005, * = *p* < 0.05).

The number of male- and female-biased genes and the magnitude of their absolute expression were not the same amongst tissues, and sometimes pointed to opposite directions. For late developmental stages (G43 and G46), female-biased genes were more numerous and had a higher absolute expression than male-biased genes (Table 1; Figure 3A,B). In gonads, however, while the absolute expression of female-biased genes was also greater, there were more male-biased genes (Table 1; Figure 3D). The opposite pattern was observed in livers and brains, with more female-biased genes, but higher expression, from the male-biased genes (Table 1; Figure 3C,E). 

We also explored sex-biased genes that showed a turnover of sex-bias in different tissues (they switched from male- to female-biased and vice versa). Across the five developmental stages, we only compared stages G43 and G46, as the first three stages had too few sex-biased genes for meaningful statistics. Interestingly, none of the 156 shared sex-biased genes between G43 and G46 showed a turnover in sex bias direction (Table 2). However, sex-bias turnover did occur among adult tissues. Amongst adult tissues, almost half of the genes (47%) showed a turnover in sex bias between gonad and liver, and 13% between gonad and brain. Finally, 6% of shared sex-biased genes between G46 and gonad showed a turnover in sex bias (Table 2). 

Gene ontology (GO) enrichment analysis of the sex-biased genes suggests that the burst of sex-biased genes at the G46 stage is probably related to gonadal development, since many GO terms are related to reproduction and are shared with gonad tissue (Supplementary Table S5). GO terms of sex-biased genes in liver and brain were not associated with reproduction, and no term of interest was detected (Supplementary Table S5). 

**Figure 4:**
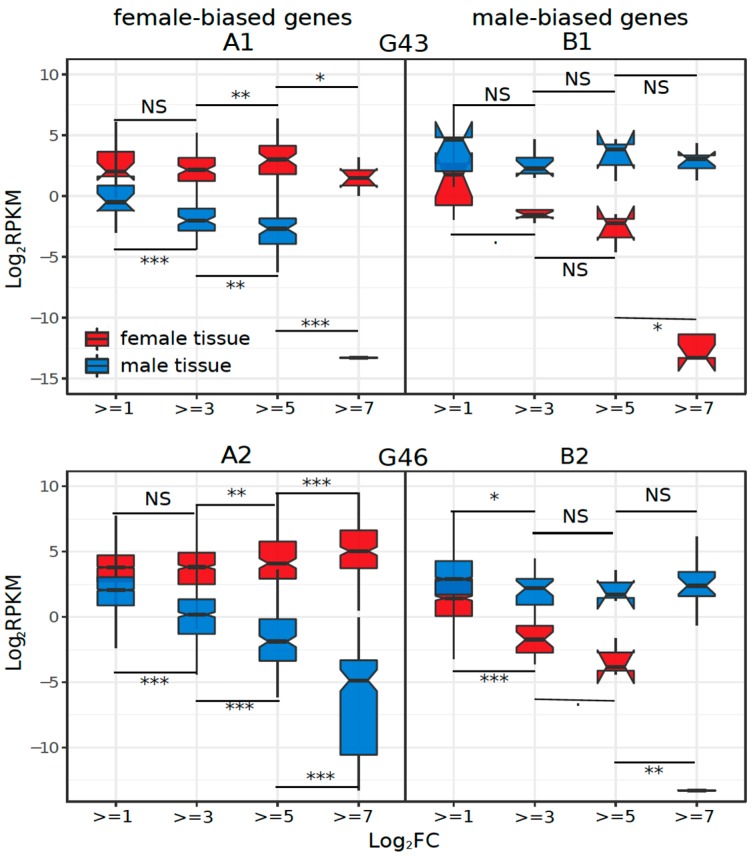

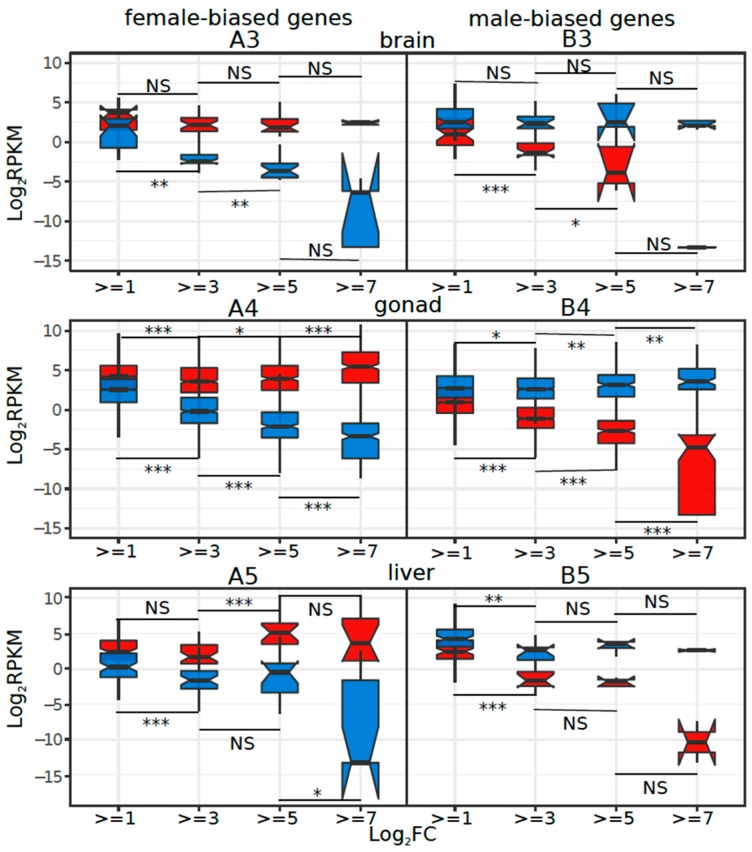
Average female- and male-biased gene expression in bins of different sex-bias fold change thresholds for (**A1**, **B1**) G43, (**A2**, **B2**) G46, (**A3**, **B3**) brain, (**A4**, **B4**) gonad, and (**A5**, **B5**) liver. The significance level of Wilcoxon rank sum tests between successive bins of male or female-biased genes is indicated above and below the boxplots (NS = non-significant, *** = *p* < 0.001, ** = *p* < 0.005, * = *p* < 0.05, . = *p* < 0.1). The statistical tests amongst negative numbers are not biologically meaningful, as all negative log_2_FC values represent raw RPKM between 0 and 1.

We investigated whether the increased sex-biased gene expression (e.g., low to high thresholds of log_2_FC) was driven by changes in male expression, female expression, or both. To do this, we binned sex-biased genes based on their log_2_FC thresholds and compared the average male and female expression in each bin for each tissue. Only the statistical results among bin comparisons for positive log_2_FC changes are biologically meaningful because they represent differences in transcript counts that might have biological consequences (all negative log_2_FC values represent raw RPKM between 0 and 1). The results show that in G43, G46, and liver, female-biased expression was caused mostly by upregulation in females (Supplementary Table S4, Figure 4(A1,A2,A5)). In contrast, in gonads, sex-biased expression was due to changes in gene expression in both sexes (Figure 4(A4)). Interestingly, in brain, both female- and male-biased gene expression were caused by downregulation in one sex (males for female-biased genes, and vice versa; Figure 4(A3), Supplementary Table S4).

We investigated the effect of sex linkage on sex bias, by examining 11,383 one-to-one orthologs between *R. temporaria* and *X. tropicalis*, for which we assigned a chromosomal position based on the strong synteny between the species [60,61,62]. We did not find a difference in frequency of male- or female-biased genes across any developmental stage or tissue on either sex chromosome (permutation test, all *p* > 0.05, Supplementary Figure S5a,b). Similarly, there were no differences between the autosomes and sex chromosomes in the log_2_ ratio of male/female expression (Wilcoxon test, all *p* > 0.05, Supplementary Figure S6). For G46 and gonad tissues, we applied a sliding window analysis of the log_2_ (male:female) gene expression ratio across chromosome length, which also did not detect an outlier peak in chromosome 1 and 2, compared to all autosomes (G46: Supplementary Figure S7; Gonad: Figure S8).

### 3.4. Coding Sequence Evolution of Sex-Biased Genes

**Figure 5:**
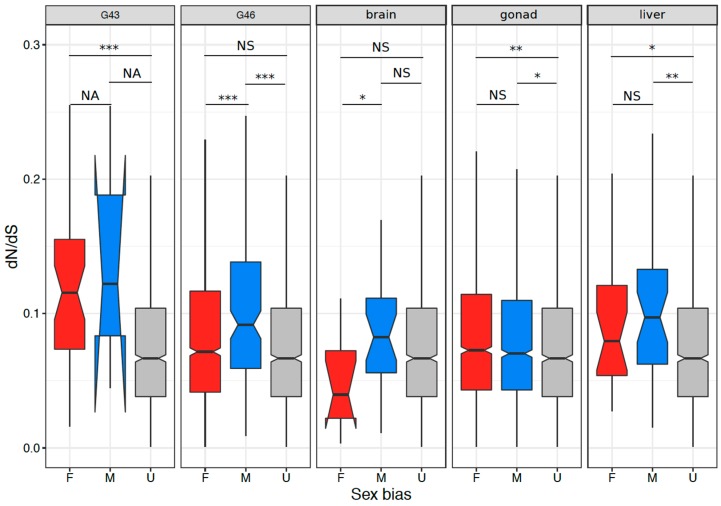
The ratios of non-synonymous to synonymous substitutions *dN*/*dS* in the tissues with >100 sex-biased genes. Significant differences based on Wilcoxon rank sum tests are denoted (NA = not applicable (due to low sample size), NS = non-significant, *** = *p* < 0.001, ** = *p* < 0.005, * = *p* < 0.05).

We compared the sequence evolution rate between sex-biased and unbiased genes that had 1:1 orthologs to *X. tropicalis*, in tissues with at least 100 sex-biased genes. We defined unbiased genes as the genes that were never sex biased in any tissue, and sex-biased genes as genes that were sex-biased in at least one tissue. The results are summarized in Figure 5 and Supplementary Table S6. Briefly, sex-biased genes tended to have a higher rate of sequence evolution than unbiased genes in all stages and tissues, except for brains (Wilcoxon test, all *p* < 0.005). In addition, male-biased genes tended to display faster sequence evolution than female-biased genes in G46 and brain (both *p* < 0.05, Supplementary Table S6). 

We further investigated the evolutionary rate of shared sex-biased genes, by comparing the average evolutionary rate of genes with and without sex-bias turnover among embryonic tissues or among adult tissues. Only pairs of embryonic tissues and adult tissues with >35 shared sex-biased genes were used. We found that genes with sex-bias turnover tended to have intermediate evolutionary rates (*dN*/*dS*) compared to consistently male-biased genes, which had the highest, and consistently female-biased genes, which had the lowest. The differences in evolutionary rates were not significant amongst these three categories of sex-biased genes between gonad and G46 tissues (Wilcoxon test, both *p* > 0.05), and between gonad and liver tissues (Wilcoxon test, both *p* > 0.2; Supplementary Figure S9a,b). 

As with sex bias, sex linkage (1169 and 987 genes from chromosome 1 and 2 respectively) did not influence the *dN*/*dS* ratio, when compared to autosomal genes (4818 genes; Supplementary Figure S10A–C). A sliding window analysis of *dN*/*dS* across chromosome length also did not detect any obvious peaks in chromosome 1 and 2, when compared to all autosomes (Supplementary Figure S11).

### 3.5. Tissue or Stage Specificity Is Highly Correlated with Sex-Biased Gene Expression and Rate of Evolution

**Figure 6:**
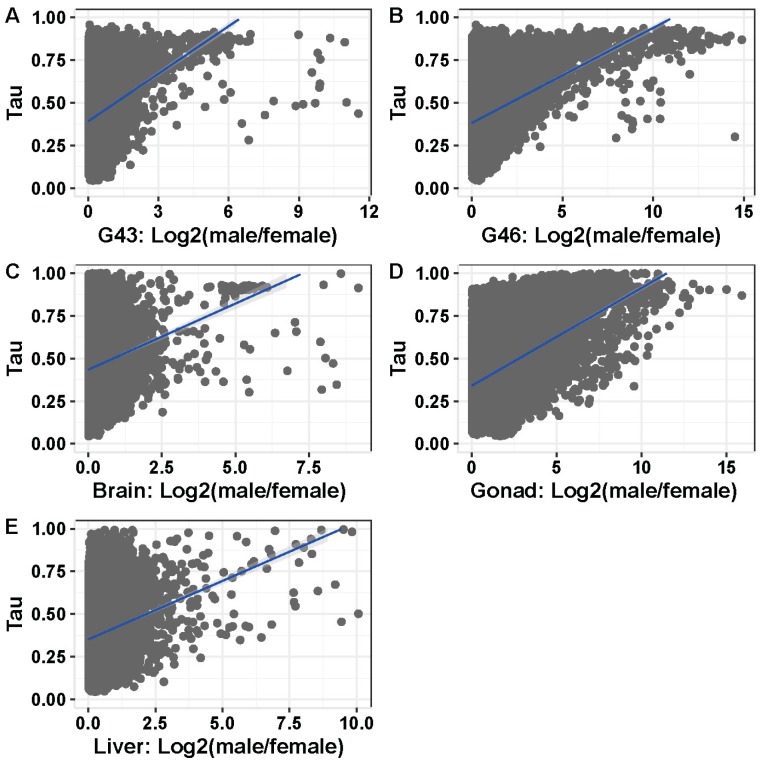
Correlation between sex-bias and tissue specificity in the five tissues with >100 sex biased genes (**A**) G43, (**B**) G46, (**C**) brain, (**D**) gonad, and (**E**) liver.

**Figure 7:**
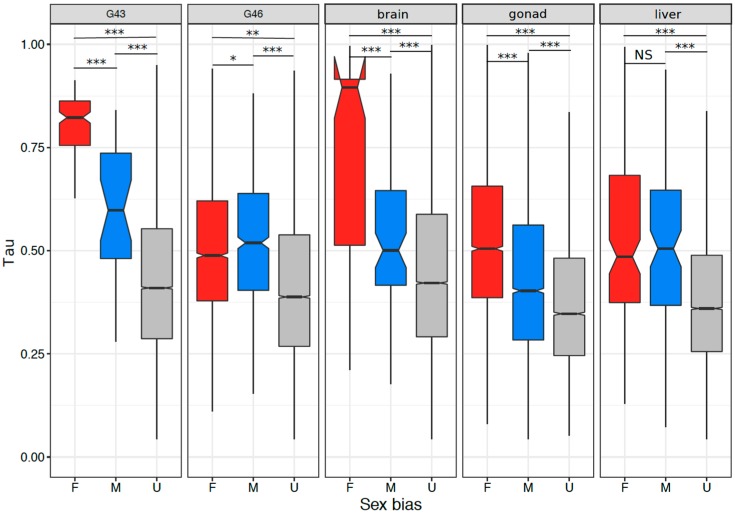
Tissue specificity in both sex-biased and unbiased genes in the five tissues with >100 sex-biased genes. Significant differences based on Wilcoxon rank sum tests are denoted (NS = non-significant, *** = *p* < 0.001, ** = *p* < 0.005, * = *p* < 0.05).

In tissues or stages with many sex-biased genes (>100), tissue specificity correlated with sex bias, and the correlation was stronger in gonads and stages containing gonads (Figure 6A–E; Spearman’s rank order correlation, G43: ρ = 0.213, *p* < 2.2 × 10^−16^; G46: ρ = 0.295, *p* < 2.2 × 10^−16^; gonad: ρ = 0.392, *p* < 2.2 × 10^−16^; brain: ρ = 0.152, *p* < 2.2 × 10^−16^; liver: ρ = 0.168, *p* < 2.2 × 10^−16^). This was also the case for samples with few sex-biased genes (G23, G27, G31; Supplementary Table S7). Both female- and male-biased genes had a significantly higher tissue specificity than unbiased genes in all analyzed tissues or stages (Figure 7, Supplementary Table S8). Furthermore, female-biased genes had a significantly higher tissue specificity than male-biased genes in G43, gonads, and brains (G43: *p* < 5.5 × 10^−10^, gonad: *p* < 2.2 × 10^−16^, brain: *p* = 1.1 × 10^−7^), but the opposite pattern was observed in G46 (*p* = 0.036). There was no difference in Tau between male- and female-biased genes in liver tissue (*p* = 0.45, Figure 7, Supplementary Table S8). When comparing the tissue specificity between shared sex-biased genes with and without sex-bias turnover, it was similar between genes with sex-bias turnover and consistently female-biased genes, and both had higher values than consistently male-biased genes (between gonad and G46 Wilcoxon test, *p* = 0.058 and *p* < 3.1 × 10^−5^ respectively; between gonad and liver tissues, *p* = 0.93 and *p* = 0.066 respectively; Supplementary Figure S9c,d).

We investigated whether the coding sequence evolution rate was best explained by tissue/stage specificity or sex bias in gene expression, by including both variables in linear models and testing whether one could be dropped. The results were dependent on tissue and developmental stage (Supplementary Table S9). In liver, gonads, and G46, there was a significant interaction between tissue specificity and sex bias (lm, all *p* < 0.05). The interaction was not significant for G43 and brains, and sex bias could be dropped from the model with data from brains (Supplementary Table S9).

## 4. Discussion

We have assembled the most comprehensive transcriptome to date for the common frog *R. temporaria*, by combining five developmental stages and three adult tissues, from a population with fully (genetically) differentiated but homomorphic sex chromosomes. This allowed us to characterize transcripts in terms of tissue specificity. We have also analyzed genes that are differentially expressed between the sexes in terms of (a) their tissue specificity, (b) their progression and turnover during embryonic development and adulthood, (c) their rate of evolution, and (d) their enrichment on sex chromosomes. In these respects, our results are largely consistent with results obtained from the same population using a transcriptome constructed from adult tissues only [53], and analysis of embryonic expression in a different population with proto-Y chromosomes [22]. Uniquely, in the present work, we have compared the influence of tissue/stage specificity and sex bias on coding sequence evolution. Below, we discuss these aspects in turn.

### 4.1. Gene Expression across Multiple Tissues and Tissue Specific Expression

The transcriptome likely includes most genes because it is assembled from a variety of stages and tissues, and hence is a valuable resource to study the dynamics of tissue-specific expression. For species lacking a reference genome, a de novo assembled transcriptome is often a good alternative; however, it is often constructed from only a few tissues (mainly gonad tissues), as in some fishes and insects [73,74]. While a transcriptome developed from one tissue is sufficient for coding sequence evolution analysis, comparisons of gene expression among tissues and characterization of tissue specificity in the expression of a gene are only possible when expression data are available from multiple tissues (e.g., [20,75,76]). We have developed a reference master transcriptome assembly in the non-model species *R. temporaria* which is more comprehensive than the previously available transcriptome from only liver tissue [77] and can be used as a common reference between studies of different *R. temporaria* populations or closely related species. The transcriptome will be publicly available as a resource to the community to study gene expression and transcriptome evolution in amphibians and vertebrates. 

Differences among tissues or stages in the transcriptome profile were greater than differences between sexes, even when considering the sexually dimorphic gonad tissues (Supplementary Figure S2). As expected, adult tissues had more distinct transcriptomic profiles than developmental stages (Supplementary Figure S2a), the later stages of which were more distinct between sexes than the early stages (Supplementary Figure S2b). This pattern of transcriptome profile grouping by tissue, and then by sex, is documented in mammals and birds spanning >300 Myr of divergence, where transcriptomic profiles cluster by organ, regardless of sex, rather than by species [78]. 

Visualization of the tissue specificity of genes with the highest expression in each tissue (Figure 1) revealed that the relative proportion of tissue specificity of highly expressed genes (top 10 and 30%) is higher in samples with reproductive tissue (G43, G46, gonad). The average tissue specificity from genes expressed in brains was always the highest, regardless of the expression level of the genes. Genes with a high expression in gonads are known to be highly tissue-specific in *Drosophila* (reviewed in [79]), mouse, and chicken specimens [14,20]. Our finding that brain tissue had the highest tissue specificity is less documented and may have been caused by the fact that brains have among the highest alternative splice variants (in humans; [80,81]). Our transcriptome was filtered to only include the highest expressed isoform per gene model; however, this process is not perfect in representing all possible splicing variants by one transcript, especially when the variants are quite distinct [13]. Future detailed investigations of alternative splicing in the common frog would be worth pursuing once a reference genome is available. 

### 4.2. Dynamics of Sex-Biased Gene Expression across Tissues and Chromosomes

Sex-biased genes increased in both number and magnitude throughout development. The early larval stages (G23, G27, G31) had very few sex-biased genes, which is consistent with only histological differences between the sexes at approximately G31 [55,56]. Most sex-biased gene expression was observed after G43, in which morphological gonad differences are visible under microscopy, suggesting that the increase in sex bias is driven by an increase in gonad tissue composition. In support, GO enrichment analysis at stage 46 found an enrichment for the term “DNA methylation involved in gamete generation” (Supplementary Table S5). Similar patterns were observed in Tvedöra, a population with proto-sex chromosomes [22], although the population studied here (Ammärnas) also showed significantly more male-biased compared to female-biased genes at stage G23 (Table 1). This difference between the populations offers the tantalizing possibility that few genes in early development are sufficient to ensure that sex-reversed (XY) individuals develop very rarely, prohibiting the male specific haplotype from recombining often (since recombination is only on very distal chromosomal regions in males [82,83]), resulting in the observed differentiation between the X and the Y. Two of these genes have homologues to *X. tropicalis* on chromosome 5 and 9. No obvious sex determining function is assigned to them, but they make interesting candidates to focus on in future studies, as do the remaining 14 male-biased genes, some of which may map to chromosome 1 or 2, but have diverged too much to identify their homologs. We cannot exclude other population specific effects on development, such as temperature, sunlight exposure, or stress for their early expression in Ammärnas. Few studies have addressed sex-biased gene expression in vertebrates at early embryonic stages, prior to the onset of gonad morphological differentiation. One such study in the rainbow trout *Oncorhynchus mykiss* (which is also an XY system with homomorphic sex chromosomes) showed a roughly equal number of male- and female-biased genes [84,85]. 

Late embryonic stages (G43, G46) showed a clear excess of female-biased genes (Table 1), a result similar to *R. temporaria* in Tvedöra [22]. This result is typical for most studies, e.g., in the clawed frog *Xenopus tropicalis* [86], chicken [3], and *Anopheles* mosquitoes [11]. Both male- and female-biased gene expression changes were driven by gene expression in female tissues (Figure 4(A1,A2,B1,B2)), suggesting that the observed patterns are primarily caused by the faster development of female reproductive tissue, which would make up a different proportion of the male and female embryos. Some of the female-biased genes might be unrelated to the gonads, but other embryonic processes instead, because there was also enrichment in GO terms unrelated to reproductive functions (Supplementary Table S9), and because 2016 (16%) sex-biased genes expressed in G46 were not sex biased in adult gonads.

Gonads had the highest sexual dimorphism in gene expression among all tissues (25%). This seems a widespread feature, with, e.g., up to 38% of transcripts in adult zebra fish [87], up to 71% of transcripts in mice [12], and up to 91% in *Drosophila* [13,17,18,19]. Gonads also had more male-biased than female-biased genes, which is often observed [13,17,18,19] and is the opposite pattern to G43, G46, liver, and brain tissues in this study. Unlike all developmental stages and livers, where gene expression changed mostly in females, sex-biased gene expression was caused by changes in expression in both sexes in gonads. Few studies have addressed the significance of which sex primarily changes expression in order to cause sex bias shifts. For instance, an increase of female-biased expression in the reproductive tissue in the dioecious willow (a plant with a ZZ/ZW system) resulted from a decrease in male tissues, suggesting that ancestral intralocus sexual conflict might have been detrimental to males, leading to the evolution of sex-biased gene expression to resolve such conflicts [88]. 

We detected very little overlap of sex-biased genes between developmental stages, confirming the rapid turnover during development previously reported for *R. temporaria* in Tvedöra [22], and other species [21]. There was also limited overlap among adult tissues in their sex-biased genes, with only 2% shared among any two tissues. Among the limited shared sex-biased genes, no turnover in sex bias direction was detected between G43 and G46, but various proportions of such events occurred in adult tissues (47% between gonad and liver, and 13% between gonad and brain). Together, the results confirm that sex-biased gene expression is highly specific to tissue and developmental stage, and care should be taken to analyze them in that context [21]. 

We did not detect the enrichment of sex-biased genes on either sex chromosome, neither in terms of the proportion of sex-biased genes, nor in terms of the relative expression intensity between males and females. If less stringent filtering is used that includes genes with high expression on G46 or gonad tissues, then an excess of female-biased genes is found on both sex chromosomes (G46: Supplementary Figure S12; Gonad: Figure S13). While the filtering we chose is recommended to avoid false positives in defining sex-biased genes, due to normal transcriptional noise in gene expression [13,89], it is interesting that this noise seems to be disproportionately mapping to the differentiated sex chromosomes. Noisy expression from the sex chromosomes may indicate ongoing changes in their gene expression in response to their disproportionate presence on one of the sexes. Differences in filtering are likely the main reason for discrepancies between the present study and one using three *R. temporaria* populations [53], although differences in the transcriptomes used (due to the tissues used in their assembly) might also play a role (e.g., in this study, we used both embryonic and adult tissues, and the others only used adult tissues). When stringent filtering criteria are used (see details in Figures S12 and S13), no enrichment in sex-biased genes is observed on the sex chromosomes in Ammarnäs, despite it representing a more advanced stage of sex chromosome evolution with most of the chromosomes 1 and 2 showing differentiation between males and females [53]. The same stringent filtering criteria were also applied in a population with proto-Y chromosomes [22], and no sex-biased gene enrichment detected at the sex chromosomes with either stringent or non-stringent filtering criteria (results not shown), further implicating sex chromosome differentiation in noisy sex-biased expression. The results suggest that sex-biased gene expression evolves after genetic differentiation, and that the increased noise in sex-biased gene expression may be an indication of ongoing gene expression evolution related to sex linkage. 

The lack of a special gene expression role for the sex chromosomes is consistent with other studies from frogs. The result is consistent with a study of juvenile stages of a *R. temporaria* population with proto-sex chromosomes (in Tvedöra) [22], the existence of sex-reversed XY adults in the wild [47,90], and the presence of within-species polymorphism and the high turnover rate of sex chromosomes in *Ranidae* [91,92,93]. It seems that an enrichment of sex-biased gene expression on the fully differentiated sex chromosomes is not required for sexual dimorphism between sexes, since it would strongly oppose such sex-chromosome transitions. In addition, in the clawed frog *Xenopus*, which has a ZW system, hybrid sterility is only determined by phenotypic sex (i.e., regardless of genotypic sex), and no differences have been found in both gene expression and fitness between normal and sex-reversed individuals [94,95]. As female-biased gene enrichment was detected in the neo-sex chromosomes of two *Drosophila* species and stickleback fish, generated by fusions between a degenerated Y and an autosome 1–15 Myr ago [9,15,45,46], the differentiated but homomorphic sex chromosomes in *R. temporaria* represent an even earlier stage of sex-chromosome evolution. More studies with various early stages of differentiated sex chromosomes are required to detect the timing when sex bias enrichment occurs. 

### 4.3. Signature of Selection on Sex-Biased Genes and Sex Linked Genes

Sex-biased genes are typically found to have a higher rate of sequence evolution through species comparisons. This is thought to reflect sex-specific evolutionary pressures acting on the loci controlling sexually dimorphic traits (reviewed in [2]). Our results largely confirm this pattern: we found that sex-biased genes had higher sequence divergence than unbiased genes in all stages and tissues except for brains. In addition, we found that male-biased genes evolved faster than female-biased genes in stage G46 and brain tissues, but not in G43, gonad, and liver tissues. A similar study in chicken specimens had more mixed results, with more varied patterns of divergence of sex-biased genes across developmental and adult stages [3]. Faster male evolution is thought to result both from stronger sexual selection [3,7,27], and from relaxed purifying selection on males (reviewed in [2]). Furthermore, we also found that sex-biased genes with a turnover in sex-bias direction in different embryonic or adult tissues tended to have intermediate evolutionary rates compared to consistently male- (the highest) and female-biased genes (the lowest). Overall, our study unveils clear signatures of sex-specific evolutionary pressures acting on sexually dimorphic traits, at late embryonic development where gonads show morphological differentiation, as well as in adult somatic (liver) and reproductive tissues. 

The *dN*/*dS* ratios of sex-linked genes on either chromosome 1 or 2 were indistinguishable from autosomal genes. These were obtained using SNPs from a transcriptome constructed from a male (X_1_X_2_Y_1_Y_2_) individual, allowing the study of the fast evolution of the Y chromosome. Previous analyses using population specific transcriptomes (XX adult tissue in [53], and combined XX and XY in a population with a proto-Y chromosome [22]) also did not detect an elevated *dN*/*dS* ratio from the sex chromosomes. The results suggest that there has been limited time for the sex chromosomes to specialize to the sex they appear the most in, even though they have been accumulating synonymous substitutions [53,83]. One possibility is that their occasional recombination through sex reversals in females counteracts their ability to retain sexually antagonistic variation [82,83], regardless of the differentiation level of the sex chromosomes (which is high in Ammärnas, [53]). We do not find enrichment in sex-biased genes, when filtering out genes with expression in a single tissue type, nor elevated rates of evolution in these differentiated sex chromosomes. Nevertheless, when genes with low and inconsistent gene expression within the same tissue type were not filtered out, the sex chromosomes showed a clear enrichment in female-biased genes, pointing to potential ongoing gene expression evolution associated with sex linkage. Taken together, our results suggest that sexually antagonistic genes, which are likely sex-biased, are not the primary drivers in anuran sex chromosome evolution, contrary to the standard models [96,97]. Instead, recombination by phenotypic sex seems to be the main factor affecting sex chromosome evolution and, while it may avoid the buildup of deleterious mutations [83,95,98], it may also slow down the accumulation of sexually antagonistic effects.

### 4.4. Rate of Evolution, Tissue Specificity, and Sex Bias

All stages and tissues showed a positive relationship between sex bias in gene expression and tissue specificity, with reproductive tissues having the highest correlation, regardless of their number of sex-biased genes. Sex-biased genes have been previously found to be less pleiotropic than unbiased genes in mouse, chicken [20], and *Drosophila* [14] specimens, suggesting that it is tissue specificity, rather than sex bias, that is responsible for the higher evolutionary rate of sex-biased genes [14]. We extended these results to amphibians, including embryonic sex bias. By analyzing sequence evolution as a function of both sex bias and tissue specificity, we found that tissue specificity is indeed more important than sex bias in explaining sequence evolution. Sex bias could be dropped from models as an explanatory variable for the rate of sequence evolution (e.g., brains), but not tissue specificity. For most developmental stages and tissues analyzed, tissue specificity and sex bias interacted in explaining sequence evolution, suggesting that sex bias contributes to the rate of sequence evolution, despite tissue specificity being more important, as previously found in mouse and *Drosophila* specimens [14]. We also found that sex-biased genes with sex-bias direction turnover tended to have similarly high Tau values to consistently female-biased genes (and higher than consistently male-biased genes), suggesting that genes with sex-bias turnover tend to be tissue specific. 

We found that overall moderately expressed genes had a higher tissue specificity than highly expressed genes across eight studied tissues or stages (Figure 1), which is consistent with the documented pattern that tissue specificity is higher for genes with a low expression [35]. By plotting the average tissue specificity of the most expressed genes in each tissue, at different thresholds of expression, we found that both brains and gonads contain comparatively highly expressed genes relative to their tissue specificity. The high expression of male-specific genes from gonads is a well-known outcome from sex-bias studies using gonads [2,16]. Highly expressed ovary and brain specific genes are less commonly described. Brains had the highest tissue specificity of all tissues. 

## 5. Conclusions

We have found that the global transcriptome profiles primarily clustered by tissue or developmental stage, and less by sex. Sex-biased gene expression increased throughout development, and gonads had the most sexually dimorphic gene expression. The reason behind increased sex-biased gene expression was either sex-specific upregulation or downregulation, or changes in gene expression in both sexes, depending on the tissue and developmental stage studied. Coding sequence evolution correlated more strongly with tissue specificity than with sex bias, but sex bias could usually not be excluded as an explanatory variable. We did not detect any enrichment in sex-biased gene expression on the sex chromosomes, but detected an excess of (few) male-biased genes in early embryonic development. Overall, the sex chromosomes do not seem to play a role in sexual development and adult sexual dimorphism despite their lack of recombination over the largest part of their length. 

## Figures and Tables

**Table 1:** Comparison of numbers of male- and female-biased genes in all studied tissues in *R. temporaria*, showing the effect of different Log_2_FC thresholds used to define sex bias. *p* values were obtained from Chi-Square tests, and *p* ≤ 0.05 is highlighted in bold. FDR: false discovery rate.

Developmental Stage/ Adult Tissue	Cut-Off Threshold (Fold Change)	Female-Biased	Male-Biased	Sex-Biased (*p* Value)
Gosner stage 23	5% FDR	4	16	0.09
	≥2	4	16	0.09
	≥4	3	16	**0.01**
	≥8	3	12	**0.05**
Gosner stage 27	5% FDR	9	2	0.066
	≥2	9	2	0.066
	≥4	9	2	0.066
	≥8	9	1	0.14
Gosner stage 31	5% FDR	11	18	0.43
	≥2	11	18	0.43
	≥4	4	14	0.16
	≥8	3	12	**0.047**
Gosner stage 43	5% FDR	156	30	**<0.0001**
	≥2	156	30	**<0.0001**
	≥4	156	30	**<0.0001**
	≥8	136	21	**<0.0001**
Gosner stage 46	5% FDR	4403	810	**<0.0001**
	≥2	3964	714	**<0.0001**
	≥4	1708	143	**<0.0001**
	≥8	802	54	**<0.0001**
Brain	5% FDR	77	83	0.82
	≥2	73	75	1
	≥4	59	44	0.36
	≥8	54	28	0.058
Liver	5% FDR	139	100	0.06
	≥2	139	100	0.06
	≥4	106	37	**<0.0001**
	≥8	68	17	**<0.0001**
Gonad	5% FDR	6059	6262	0.2
	≥2	5227	5687	**<0.001**
	≥4	2538	2901	**<0.0001**
	≥8	1317	1260	0.44

**Table 2:** Number of sex-biased gene turnovers in their sex bias direction, between tissues with >35 shared sex-biased genes.

Stage	Tissue	Nr. Shared Sex-Biased Genes	Turnover in Sex Bias Direction		Proportion of Turnover in Total Sex Bias
			Female to Male	Male to Female	
Juvenile	G43 vs. G46	156	0	0	0%
Adult	Gonad vs. Liver	92	15	28	47%
	Gonad vs. Brain	62	7	1	13%
Juvenile and Adult	G46 vs. Gonad	2661	134	29	6%

## Supplementary Materials

The following are available online at http://www.mdpi.com/2073-4425/9/6/294/s1, Figure S1: Multidimensional scaling plot of gene expression data among samples within each tissue, (A) G23, (B) G27, (C) G31, (D) G43, (E) G46, (F) gonad, (G) brain, (H) liver, Figure S2: Principle component analysis of gene expression data among all eight different tissues. (A) PC1 vs. PC2, (B) PC3 vs. PC4, (C) PC5 vs. PC6, Figure S3: Venn diagram of sex-biased genes among (A) five developmental stages, (B) three adult tissues, Figure S4: Summary of sex-biased genes defined by different fold-change threshold across five developmental stage tissues (G23, G27, G31, G43, G46), and three adult tissues (brain, gonad, liver), Figure S5: Permutation test for chromosome enrichment compared to autosomal genes for (A) female-biased genes, (B) male-biased genes, Figure S6: Wilcoxon tests for Log_2_FC gene expression ratio differences between sex-linked and autosomal genes for five developmental stages and three adult tissues, Figure S7: In G46 tissue, gene expression ratio Log_2_(male:female) over the length of sex chromosomes (Chromosome 1, and Chromosome 2), as well as each of 8 autosomes with sliding window of 40 genes, Figure S8: In gonad tissue, gene expression ratio Log2(male:female) over the length of sex chromosomes (Chromosome 1, and Chromosome 2), as well as each of 8 autosomes with sliding window of 40 genes, Figure S9: *dN*/*dS* ratios (a,b) and tissue specificity (c,d) of genes with turnover in sex-bias direction, and sex-biased genes being consistent in sex-bias direction (female, male respectively) between gonad and G46 tissues (a,c), and between gonad and liver tissues (b,d), Figure S10: Wilcoxon tests for differences in synonymous mutations, non-synonymous mutations and their ratio between the autosomes and sex chromosomes (A–C), Figure S11: *dN*/*dS* ratios over the length of sex chromosomes (Chromosome 1, and Chromosome 2), as well as each of 8 autosomes with sliding window of 40 genes, Figure S12: Permutation test for chromosome enrichment compared to autosomal genes for female (A,C,E), male-biased genes (B,D,F), as well as total Log_2_(male:female) gene expression ratio in stage G46 tissue, generated under different filtering criteria. (A,G) (total Log_2_CPM > 2), (C,H) (average Log_2_CPM > 0) are under relaxed filtering criteria, while (E) is from stringent filtering criteria (average Log_2_CPM > 0, and CPM > 1 in at least half of the samples per sex), Figure S13: Permutation test for chromosome enrichment compared to autosomal genes for female (A,C,E), male-biased genes (B,D,F), as well as total Log_2_(male:female) gene expression ratio in gonad tissues, generated under different filtering criteria. (A,G) (total Log_2_CPM > 2), (C,H) (average Log_2_CPM > 0) are under relaxed filtering criteria, while (E) is from stringent filtering criteria (average Log_2_CPM > 0, and CPM > 1 in at least half of the samples per sex), Table S1: *Dmrt1* genotyping of all samples used in RNAseq analysis and the indicated genotypic sex, Table S2: Genomic distributions of one-to-one orthologs of reciprocal best blast hit between *Rana temporaria* and *Xenopus tropicalis*, Table S3: Summary of super exact tests for overlap of sex biased genes between any two of the five developmental stages, Table S4: Wilcoxon non-parametric test between sex-biased and unbiased gene expression in both female and male tissues, as well as comparison among sex-biased gene expression of various Log_2_FC cut off for G43, G46, gonad, brain and liver tissues, Table S5: GO terms of sex-biased genes for G46 and three adult tissues, Table S6: Wilcoxon rank sum test for rate of evolution (*dN*/*dS*) among female-biased, male-biased, and unbiased genes in tissues with >100 male biased genes, Table S7: Spearman’s rank correlation test between Log_2_(male/female) gene expression ratio and tissue specificity in tissues with <100 sex biased genes, such as G23, G27, G31 tissues, Table S8: Wilcoxon rank sum test for tissue specificity among female-biased, male-biased, and unbiased genes in tissues with >100 sex biased genes, Table S9: Summary of linear models testing the effects of sex bias, tissue specificity and their interaction in the rate of coding sequence evolution, Text S1: Method description of producing a de novo transposable element library from *Rana temporaria*.

## Founding

This work was supported by the Swiss National Science Foundation (grants 31003A_166323 to N.P.; and Sinergia grant CRSII3_147625 to N.P., John Pannell and Mark Kirkpatrick).

## Availability of Data and Materials

We have deposited the raw reads in NCBI Sequence Read Archive (SRA) with the accession SRP149620 (SRR7251158-SRR7251189 and SRR7263262-SRR7263290) under BioProject ID PRJNA474233. Scripts related to the analysis presented here, and the repeats library, are archived at DOI: 10.5281/zenodo.1258010 (GitHub repository: https://github.com/Wen-Juan/Rana_Transcriptome).
