# Supplementary material for: Tissue Specificity and Dynamics of Sex-Biased Gene Expression in a Common Frog Population with Differentiated, Yet Homomorphic, Sex Chromosomes
Source: Genes (Basel). 2018 Jun 12;9(6):294. doi: 10.3390/genes9060294 (PMC6027210; doi:10.3390/genes9060294)
Supplement: Supplementary file 1 [file genes-09-00294-s001.zip › all_suppl/suppl_tables/TextS1.docx]

**Methods**

Anuran genomes are known to contain high numbers of active transpose elements (Sun et al. 2015), which could add substantial noise to transcriptomic data. Thus, we endeavoured to mask as much of this signal as possible prior to analyses. We first produced a *de novo* repeat library using dnaPipeTE (Goubert et al. 2015) and an existing short-read whole genome sequencing dataset (Brelsford et al. 2016). DnaPipeTE works under the assumption that, when given a low coverage sequence dataset (<1x coverage), assembly of reads into contigs should only be successful for sequence which is present many times in the genome (i.e. repeats). Due to their high copy number in DNA extractions, it was first necessary to filter mitochondrial sequences from the dataset, which would otherwise occur as false positives in the final repeat library. The full mitochondrial genome for the sequenced individual was assembled from the short-read dataset using mitoBIM (Hahn et al. 2013) using a full *cytb* gene sequence (Gene ID: 3283504) as the seed. The dataset was then aligned to the assembled mitochondrial genome using Blastn, and reads successfully aligned were removed. Using dnaPipeTE, we then subsampled the mitochondria-filtered dataset (~50x coverage) to a depth of 0.2x coverage using a genome size estimate of 4.5 Gb. These reads were then subjected to sequential rounds of assembly and the resulting contigs were annotated against existing repeat libraries.

**References**

Brelsford, A., Rodrigues, N., Perrin, N. 2016. High-density linkage maps fail to detect any genetic component to sex determination in a *Rana temporaria* family.

Goubert, C. et al., 2015. De Novo Assembly and Annotation of the Asian Tiger Mosquito (Aedes albopictus) Repeatome with dnaPipeTE from Raw Genomic Reads and Comparative Analysis with the Yellow Fever Mosquito (Aedes aegypti). *Genome biology and evolution*, 7(4), pp.1192–1205.

Hahn, C., Bachmann, L. & Chevreux, B., 2013. Reconstructing mitochondrial genomes directly from genomic next-generation sequencing reads--a baiting and iterative mapping approach. *Nucleic acids research*, 41(13), p.e129.

Sun, Y.-B. et al., 2015. Whole-genome sequence of the Tibetan frog *Nanorana parkeri* and the comparative evolution of tetrapod genomes. *Proceedings of the National Academy of Sciences of the United States of America*, 112(11), pp.E1257–62.
