# Supplementary figures and images for: Tissue Specificity and Dynamics of Sex-Biased Gene Expression in a Common Frog Population with Differentiated, Yet Homomorphic, Sex Chromosomes
Source: Genes (Basel). 2018 Jun 12;9(6):294. doi: 10.3390/genes9060294 (PMC6027210; doi:10.3390/genes9060294)

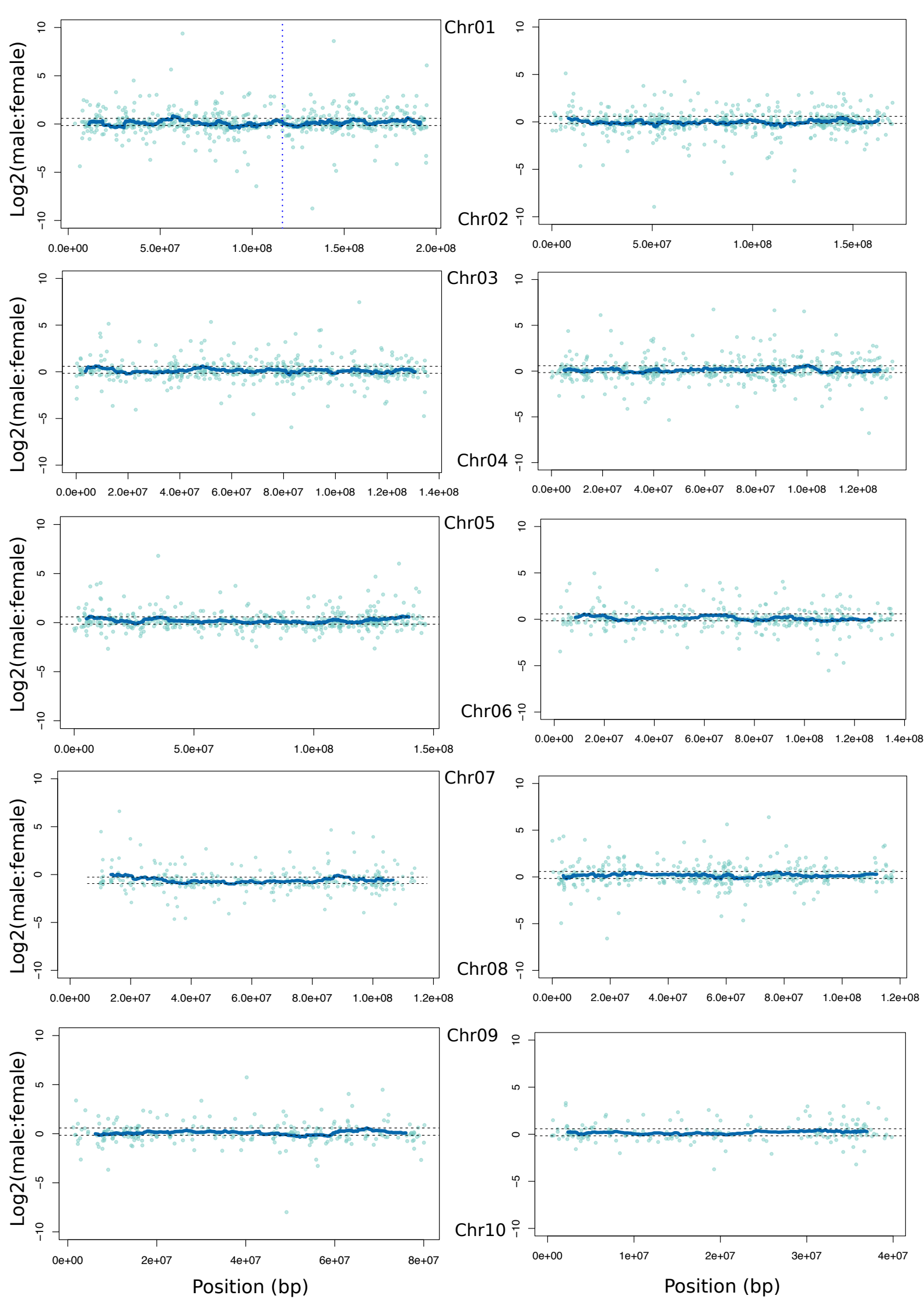

Supplement: Supplementary file 1 [file genes-09-00294-s001.zip › all_suppl/suppl_figures/FigureS8.pdf]

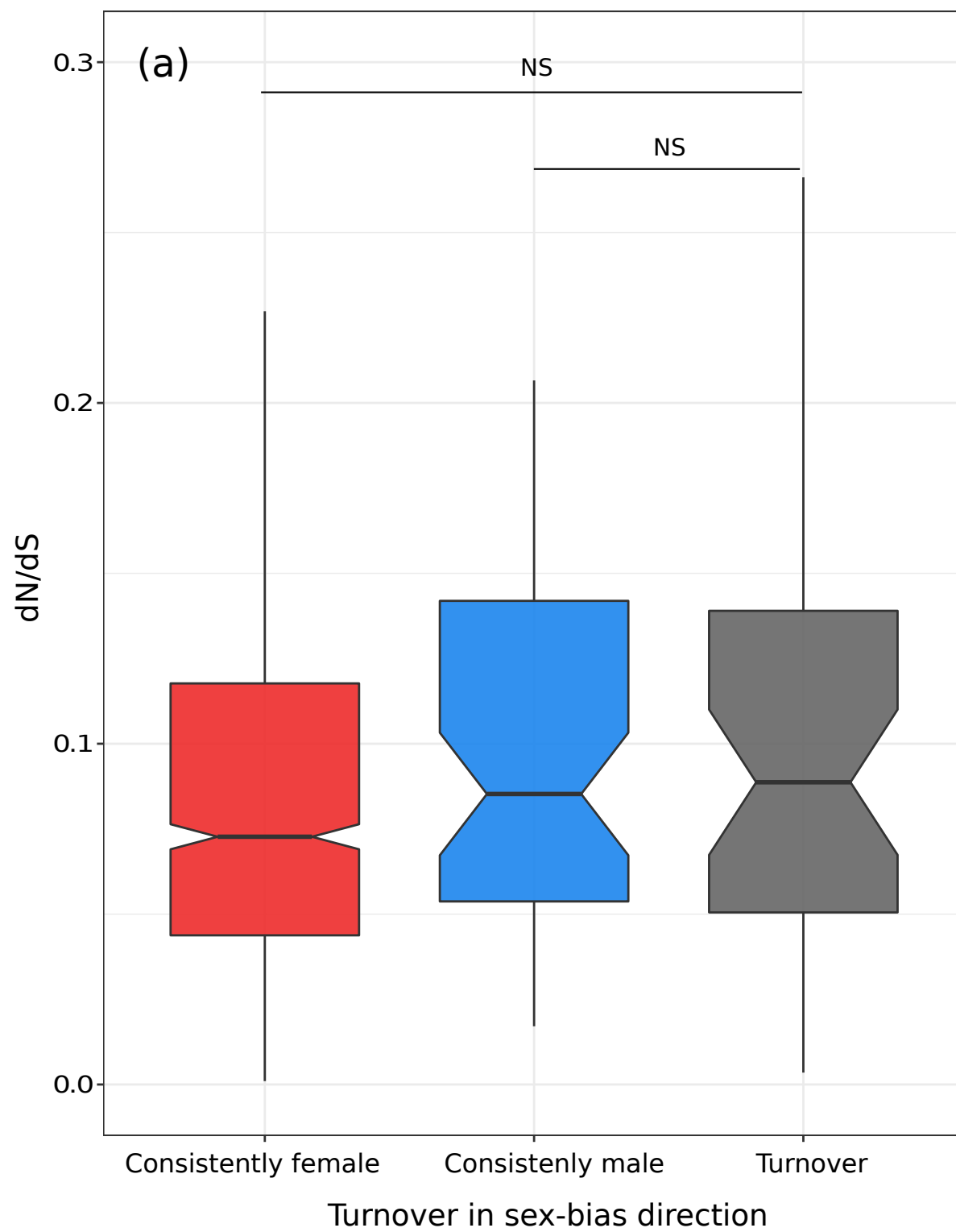

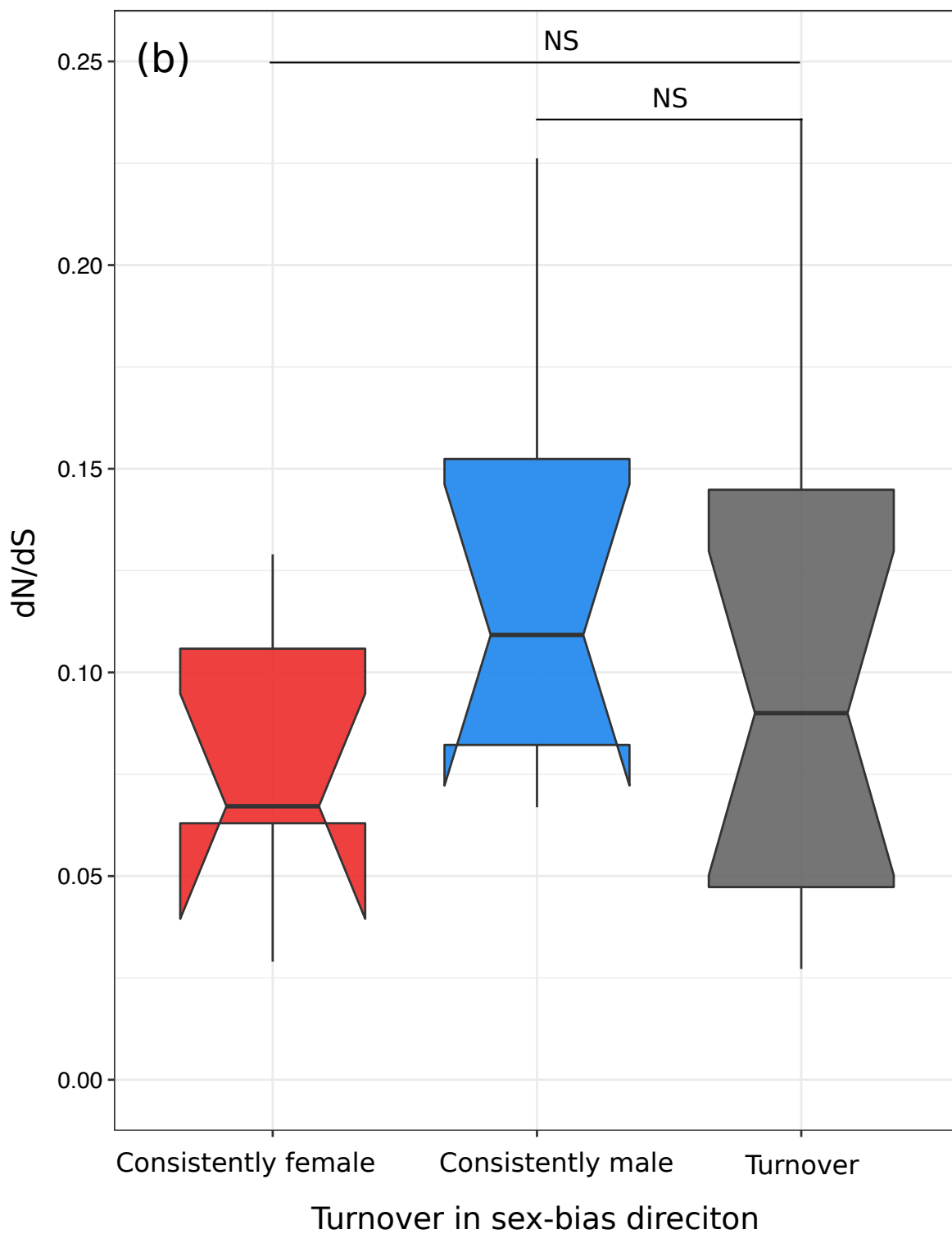

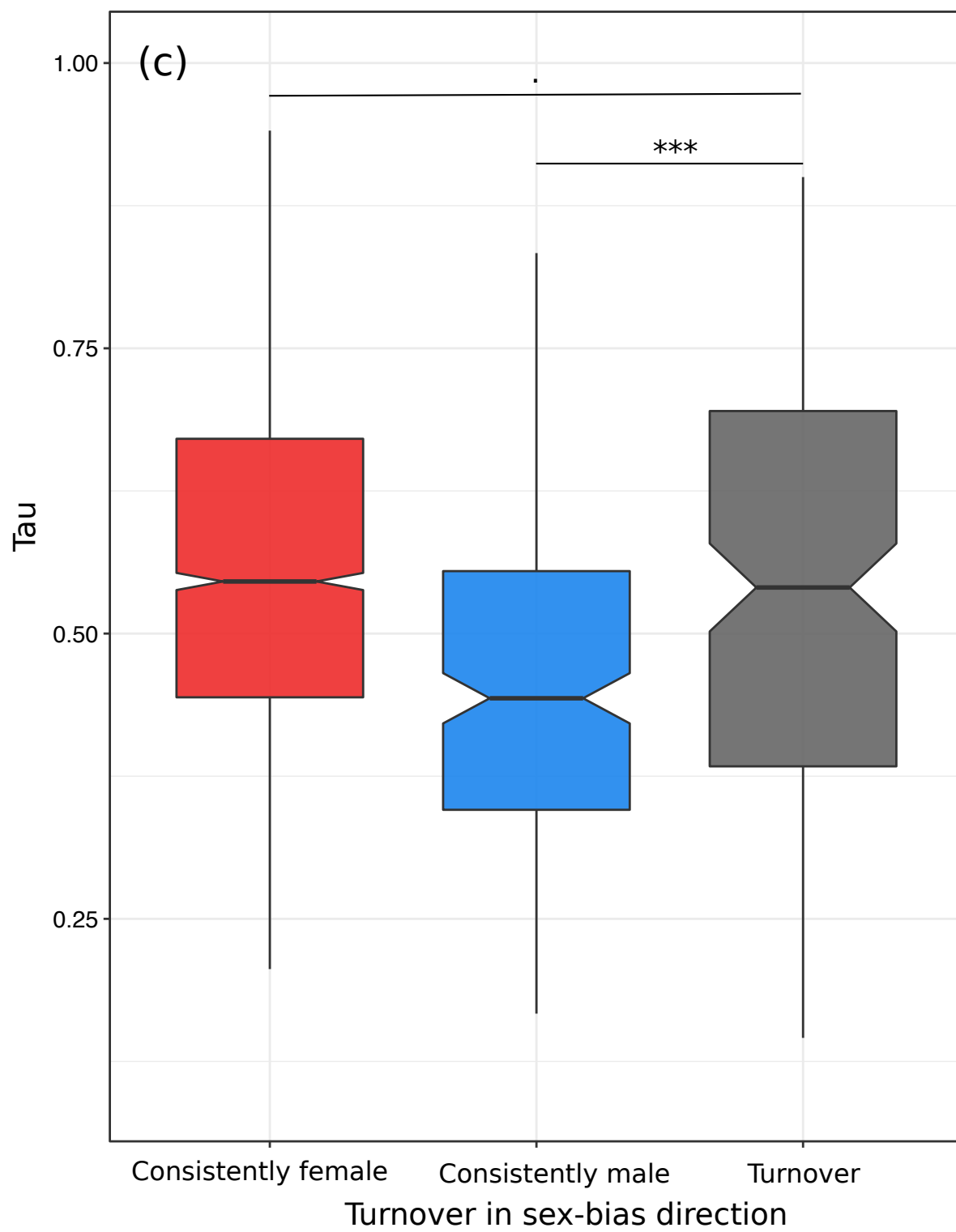

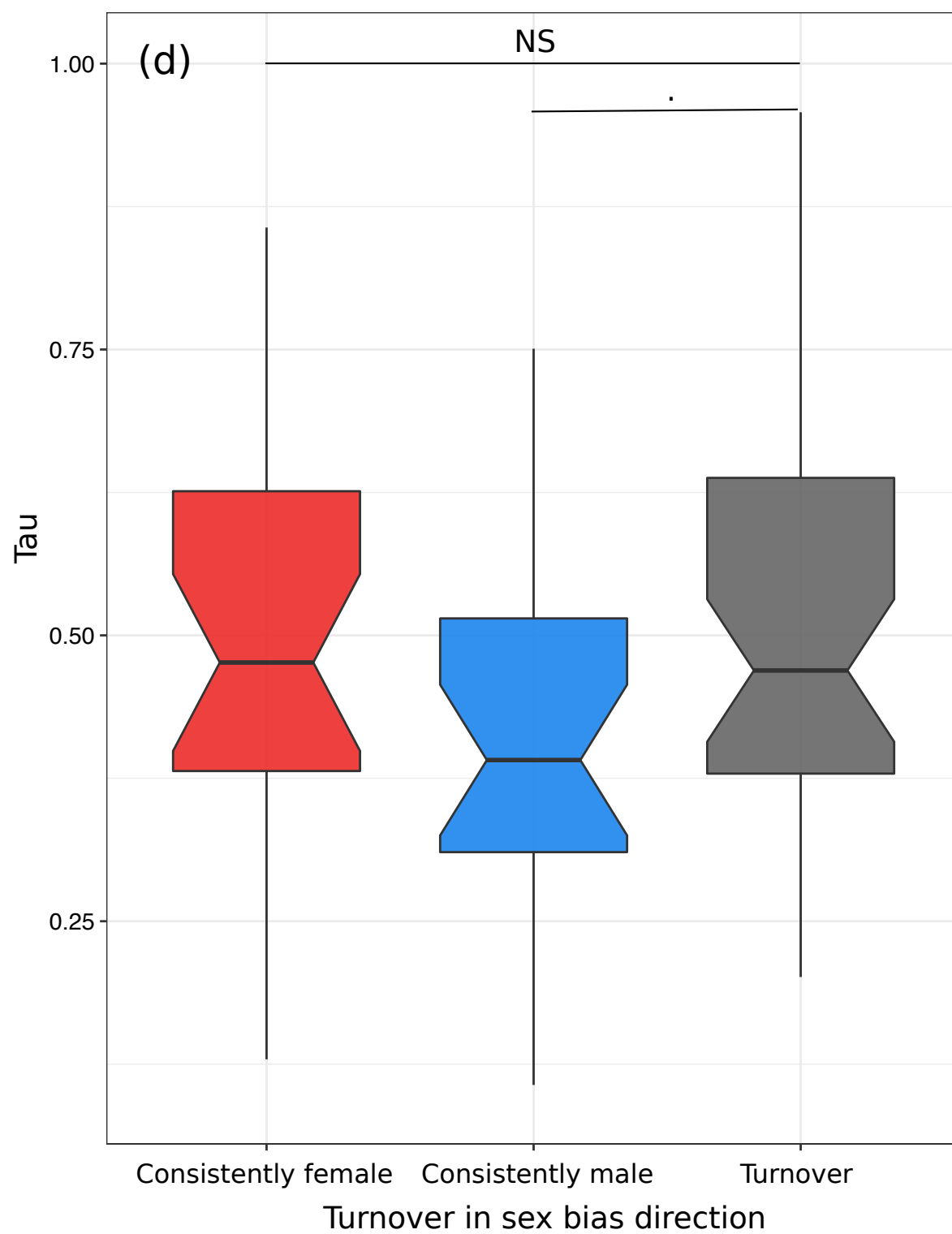

Supplement: Supplementary file 1 [file genes-09-00294-s001.zip › all_suppl/suppl_figures/FigureS9.pdf]

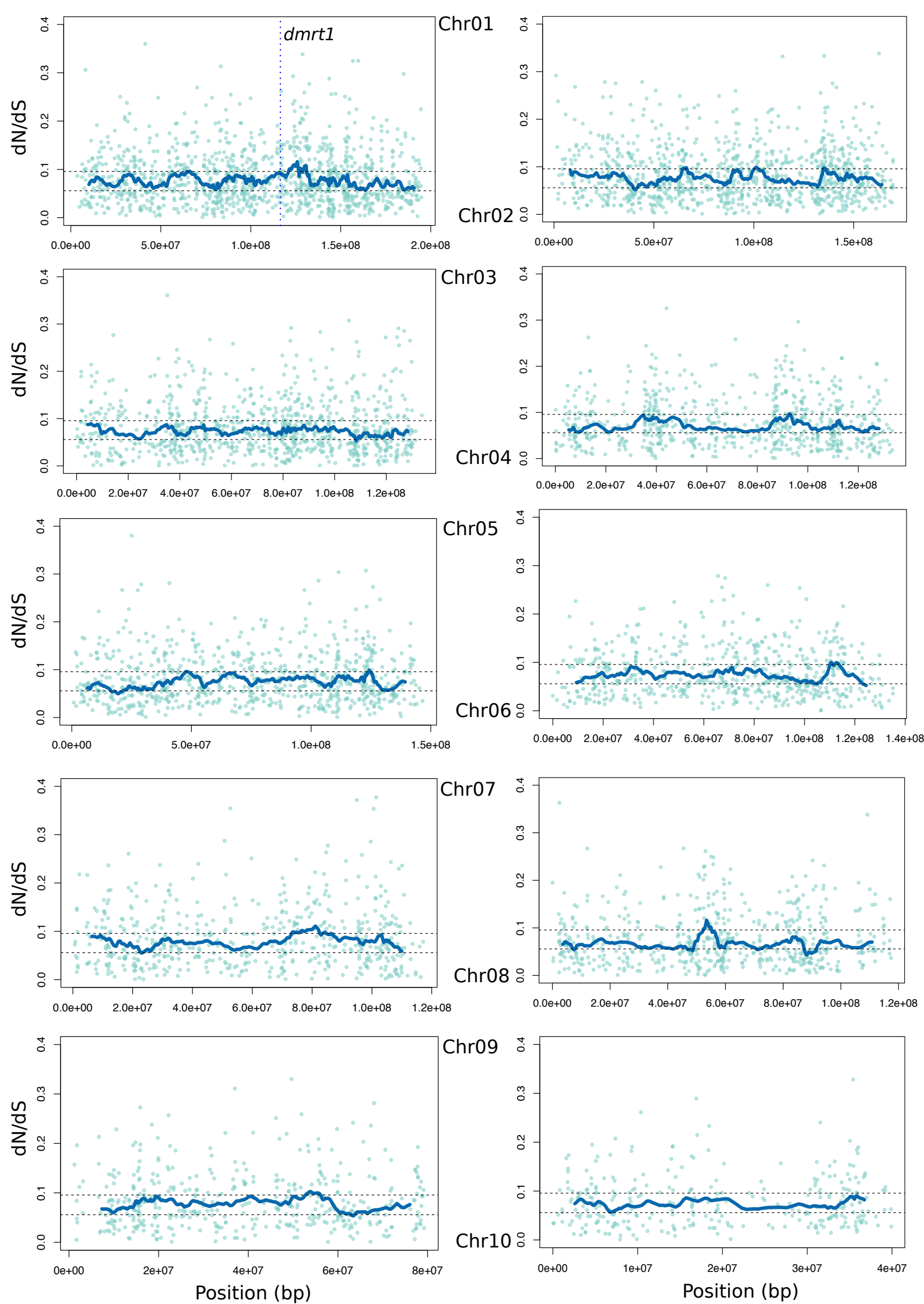

Supplement: Supplementary file 1 [file genes-09-00294-s001.zip › all_suppl/suppl_figures/FigureS11.pdf]

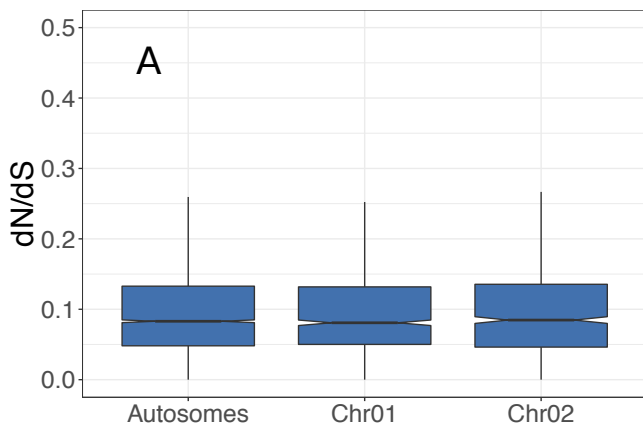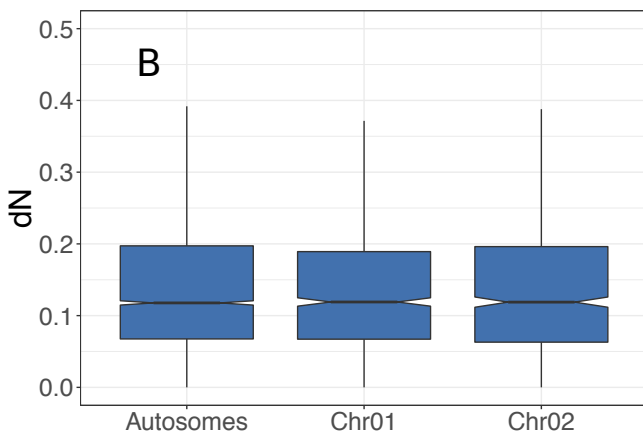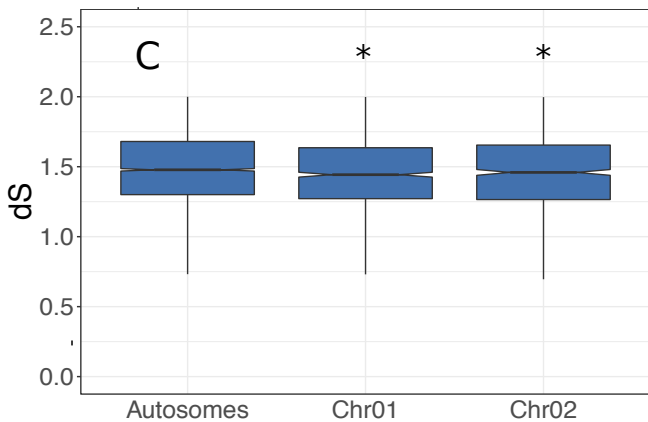

Supplement: Supplementary file 1 [file genes-09-00294-s001.zip › all_suppl/suppl_figures/FigureS10.pdf]

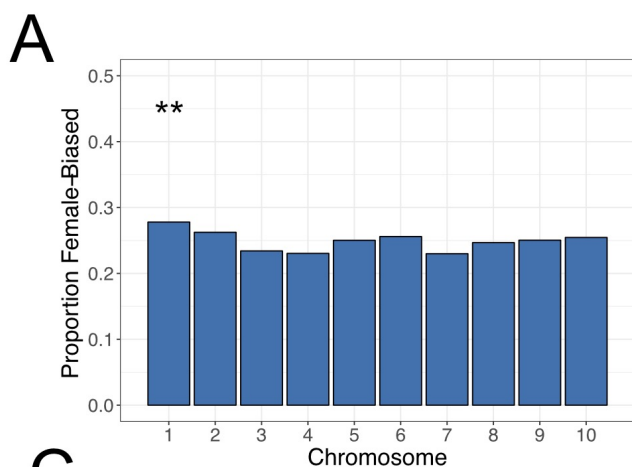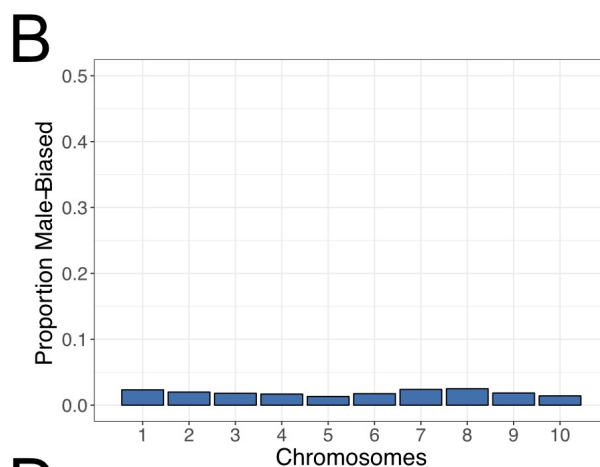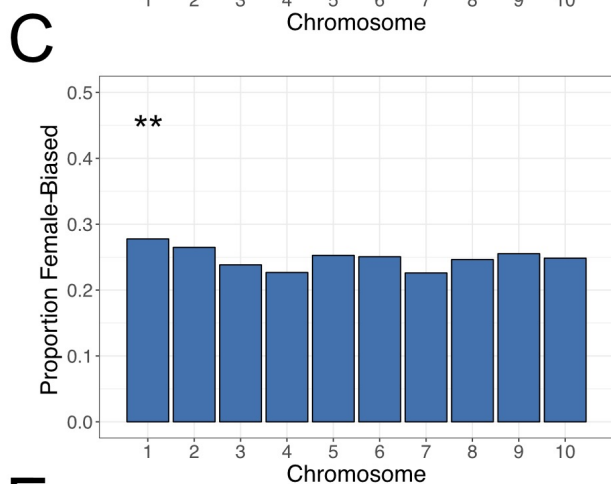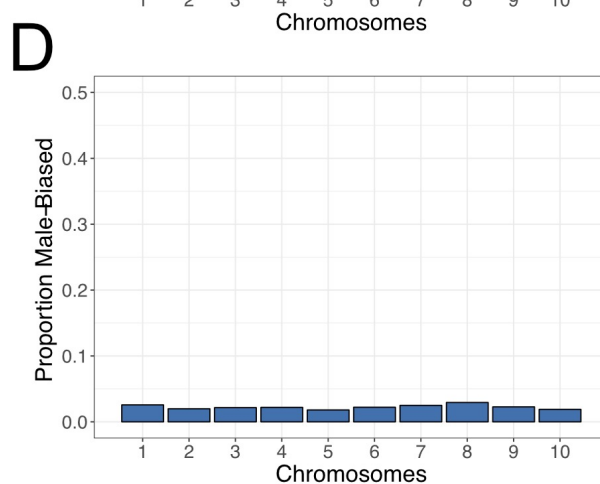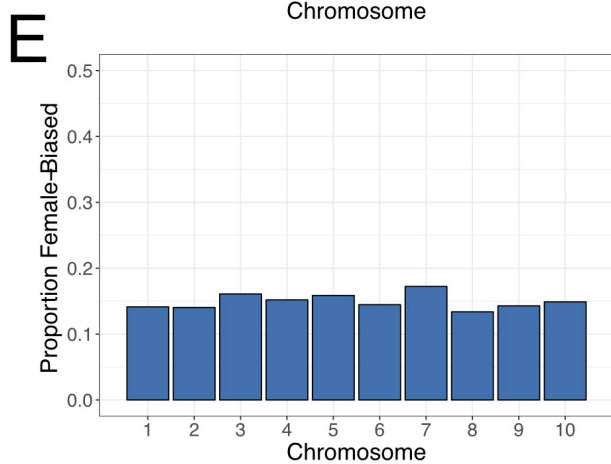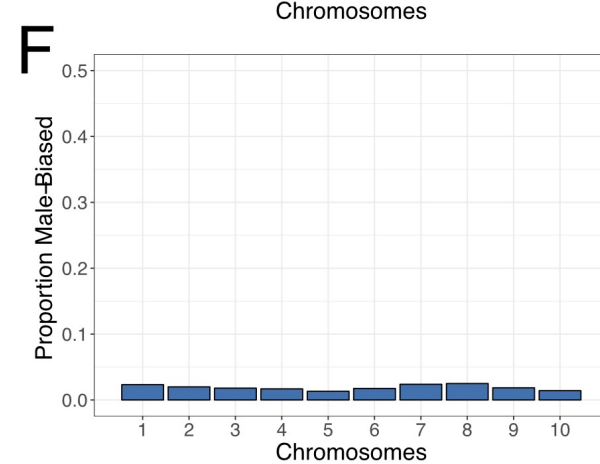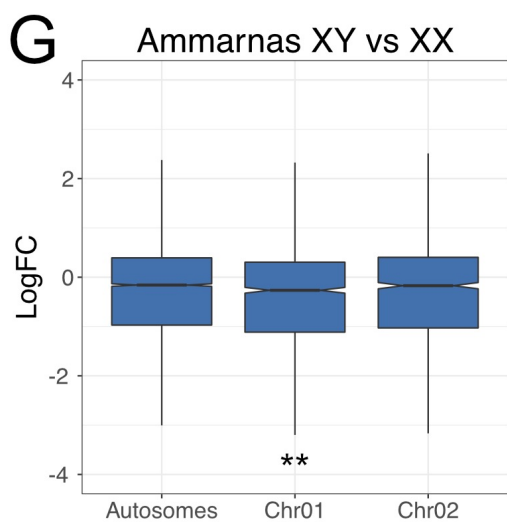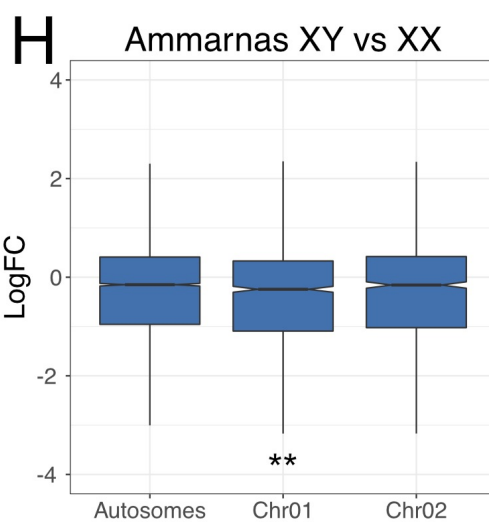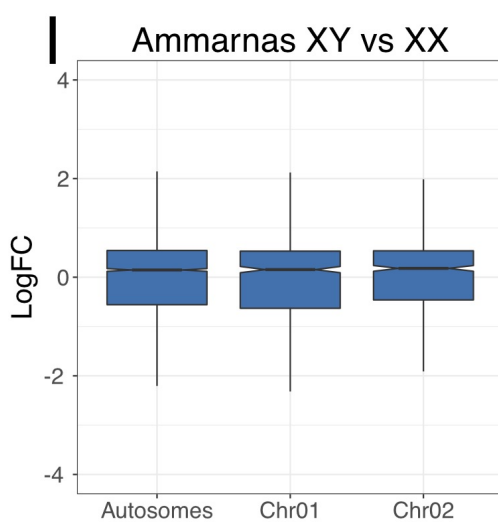

Supplement: Supplementary file 1 [file genes-09-00294-s001.zip › all_suppl/suppl_figures/FigureS12.pdf]

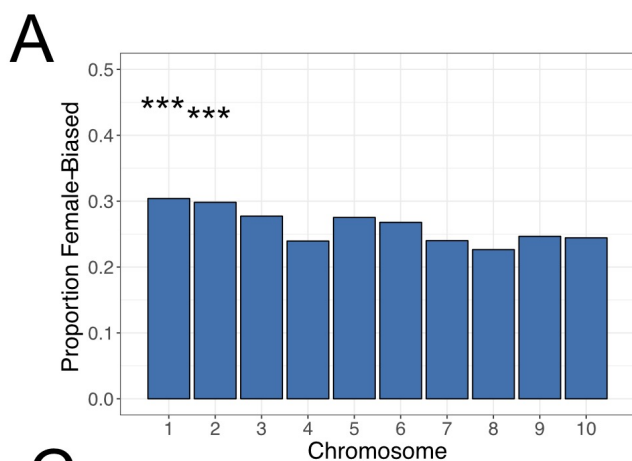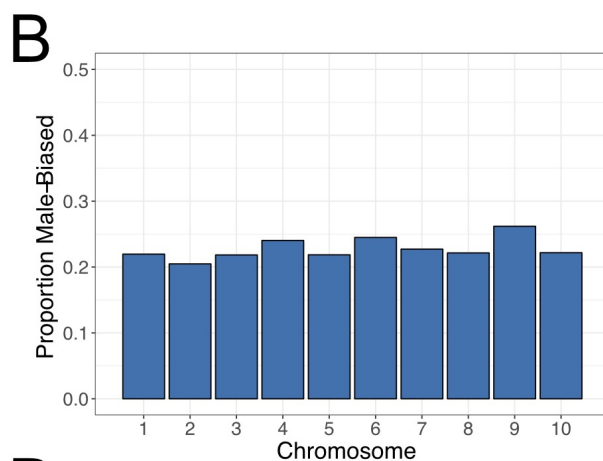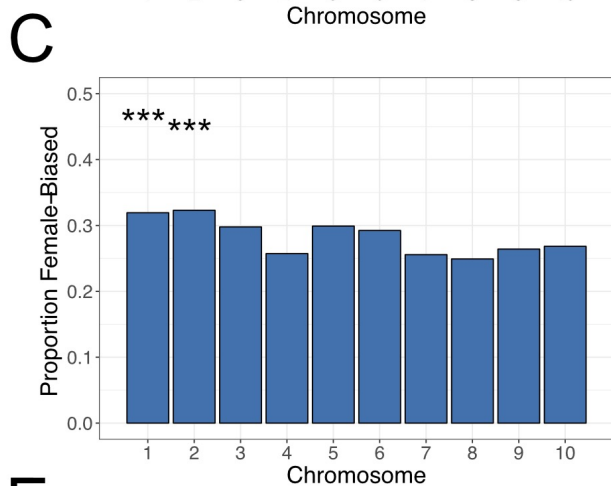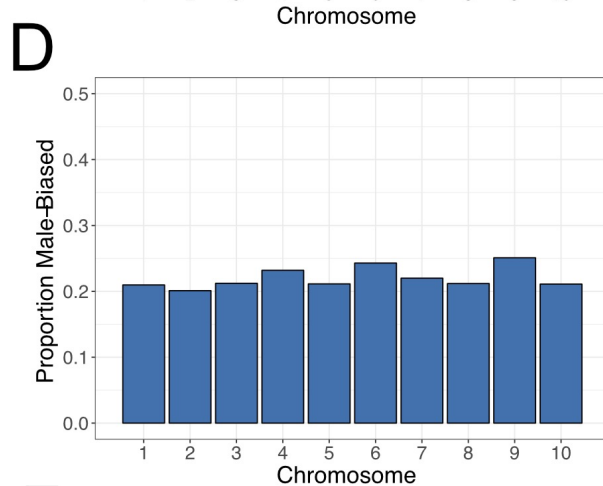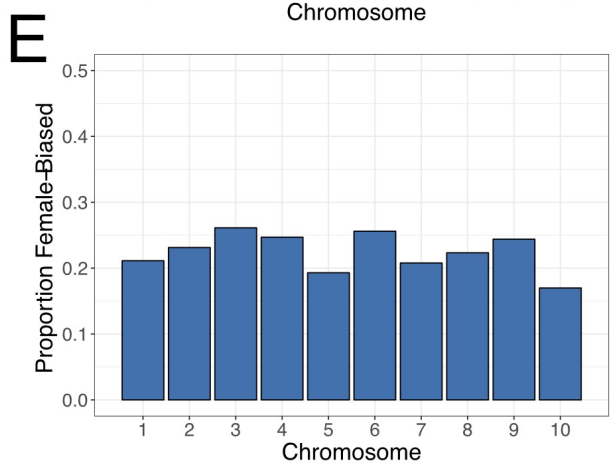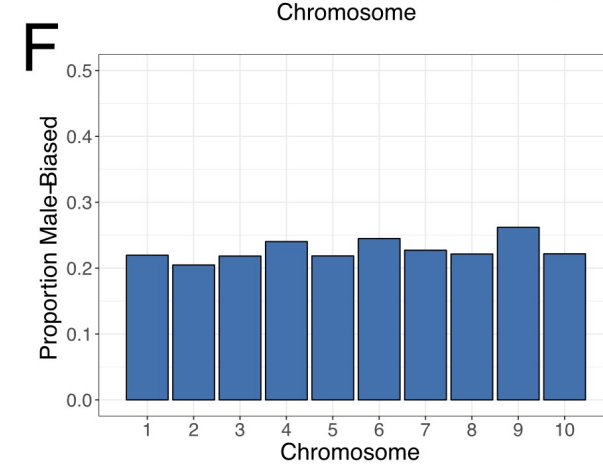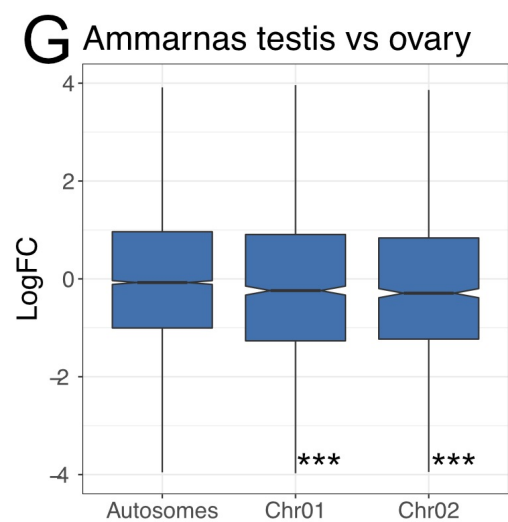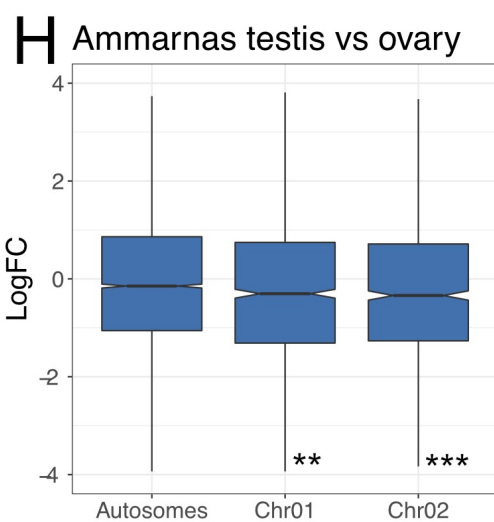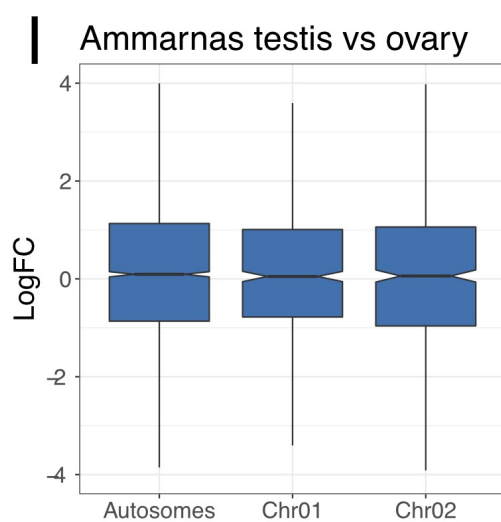

Supplement: Supplementary file 1 [file genes-09-00294-s001.zip › all_suppl/suppl_figures/FigureS13.pdf]

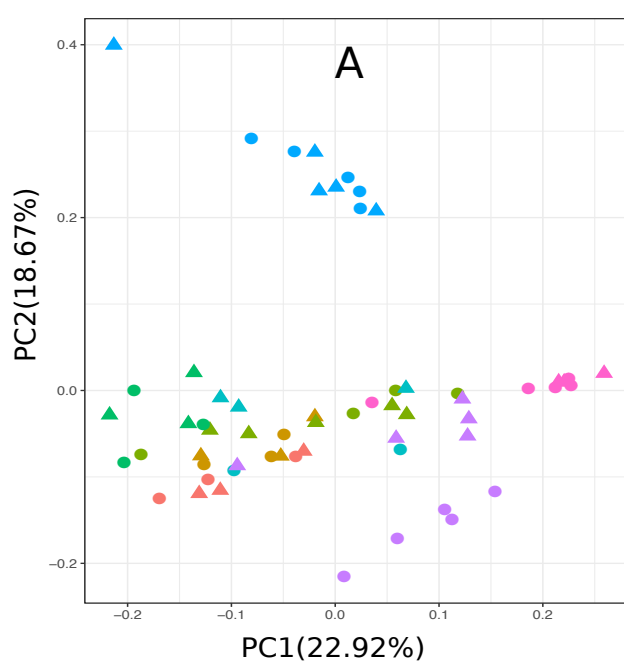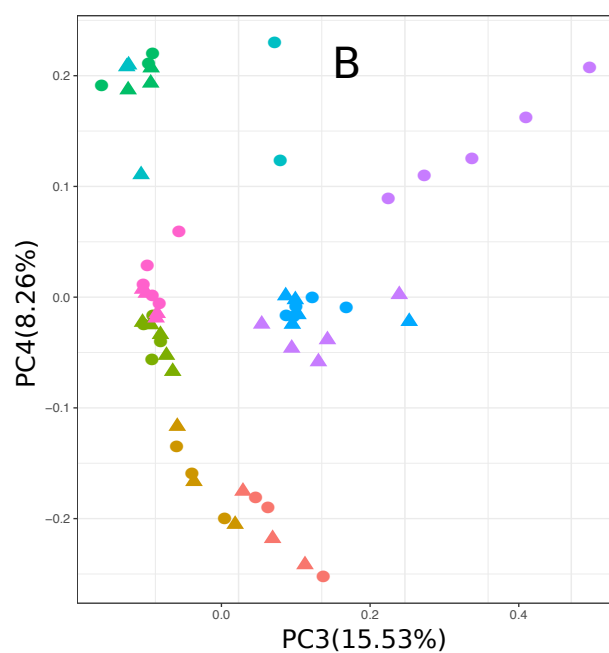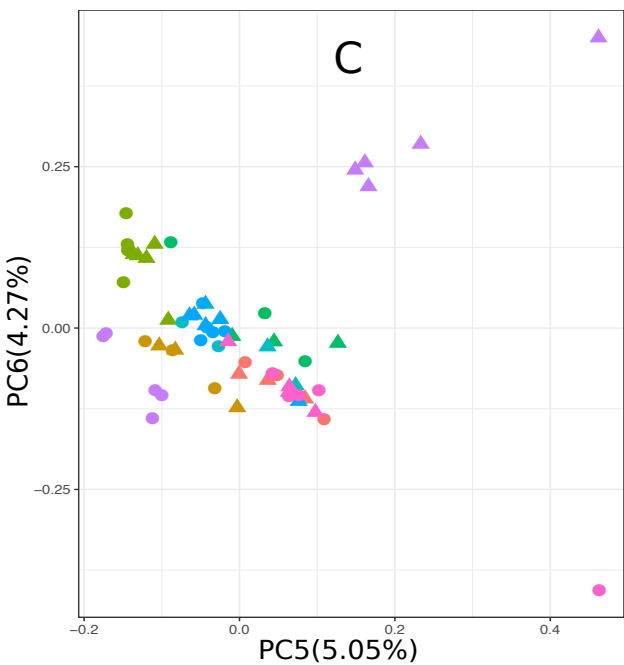

Supplement: Supplementary file 1 [file genes-09-00294-s001.zip › all_suppl/suppl_figures/FigureS2.pdf]

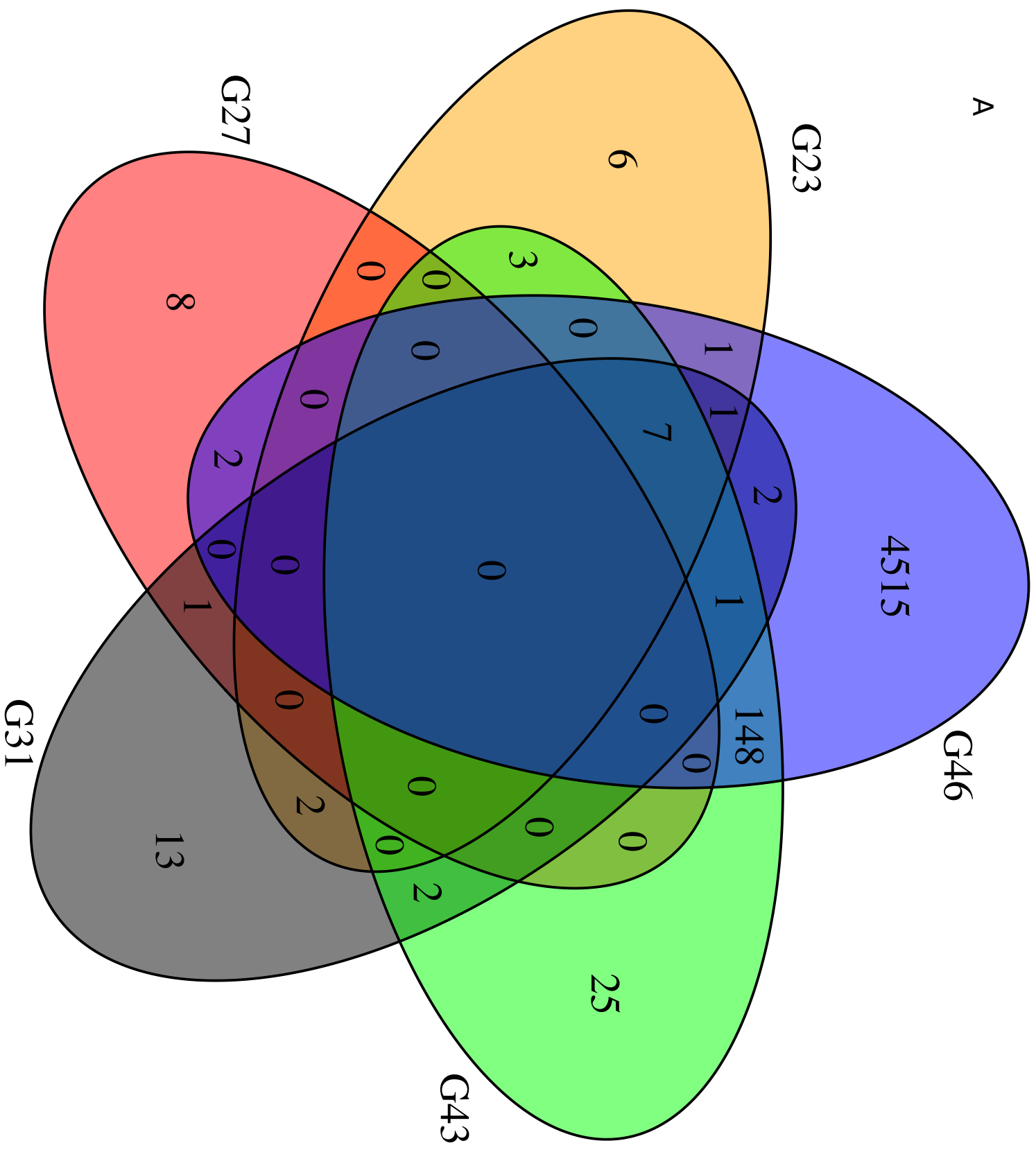

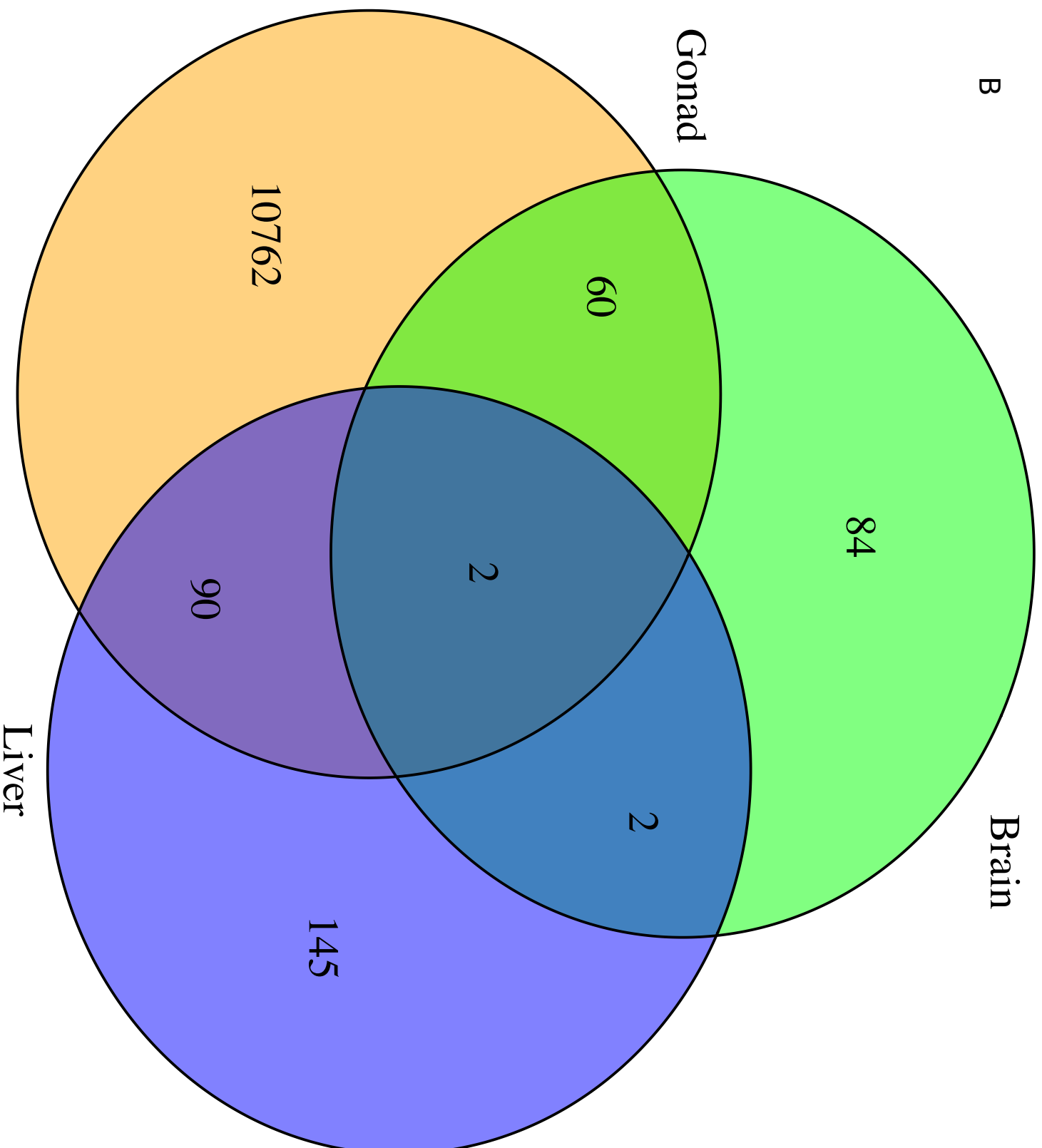

Supplement: Supplementary file 1 [file genes-09-00294-s001.zip › all_suppl/suppl_figures/FigureS3.pdf]

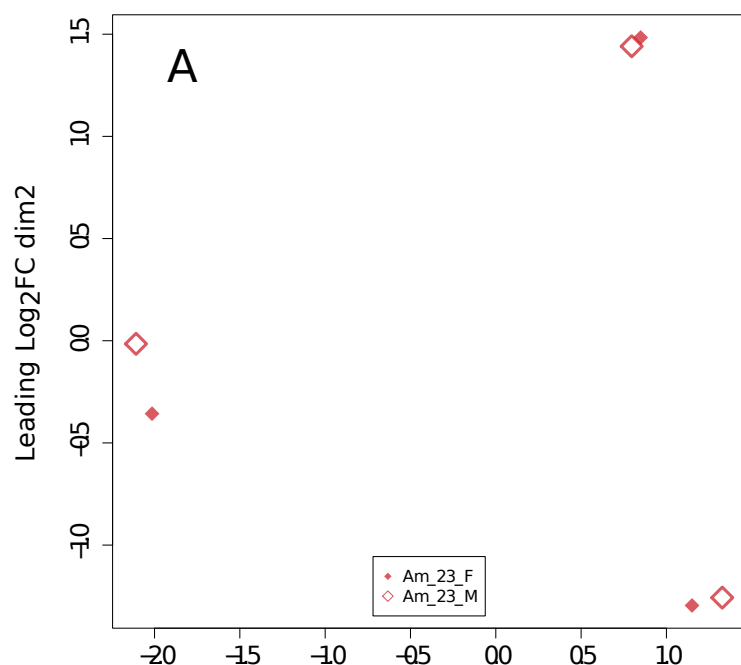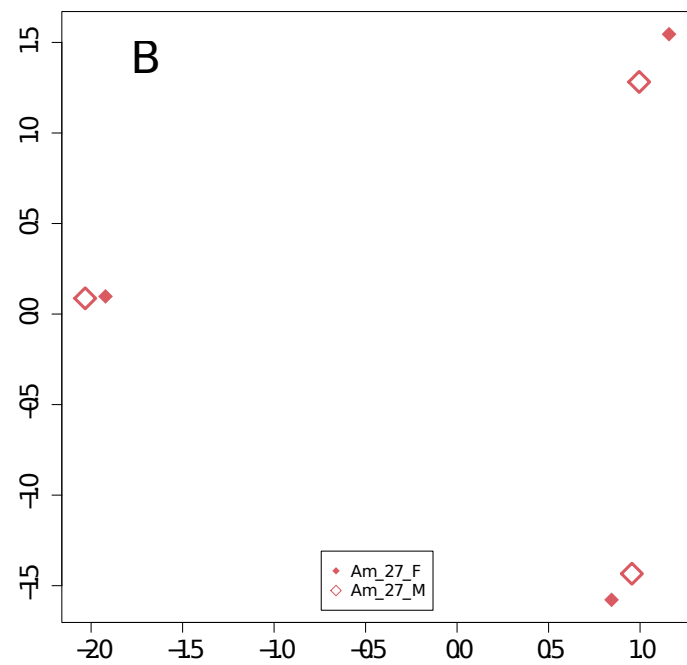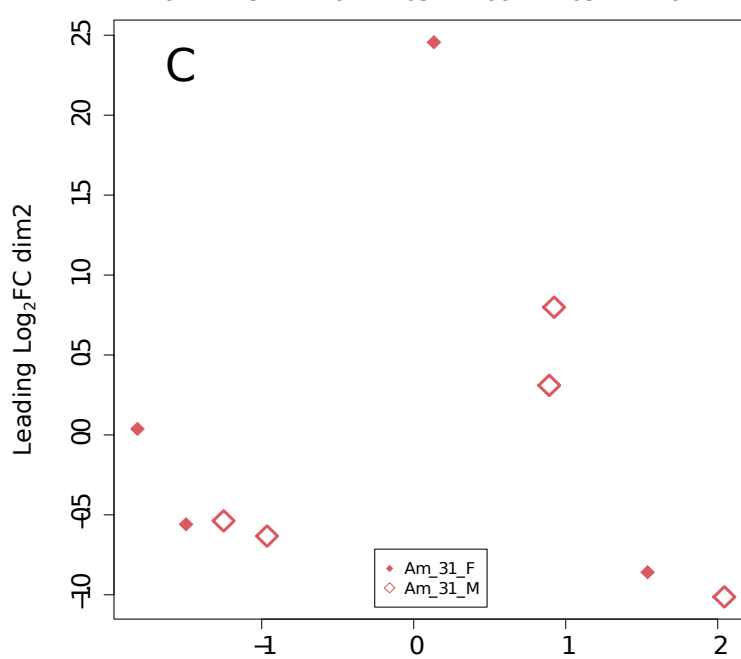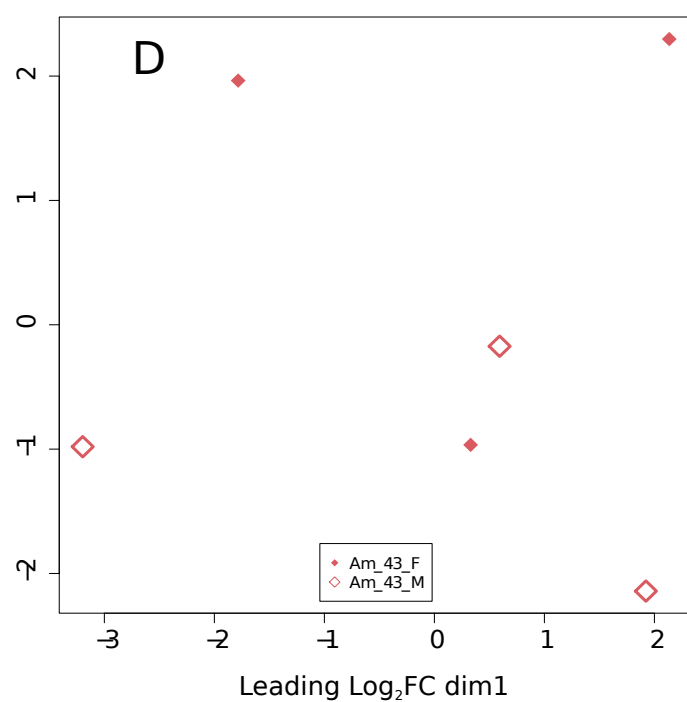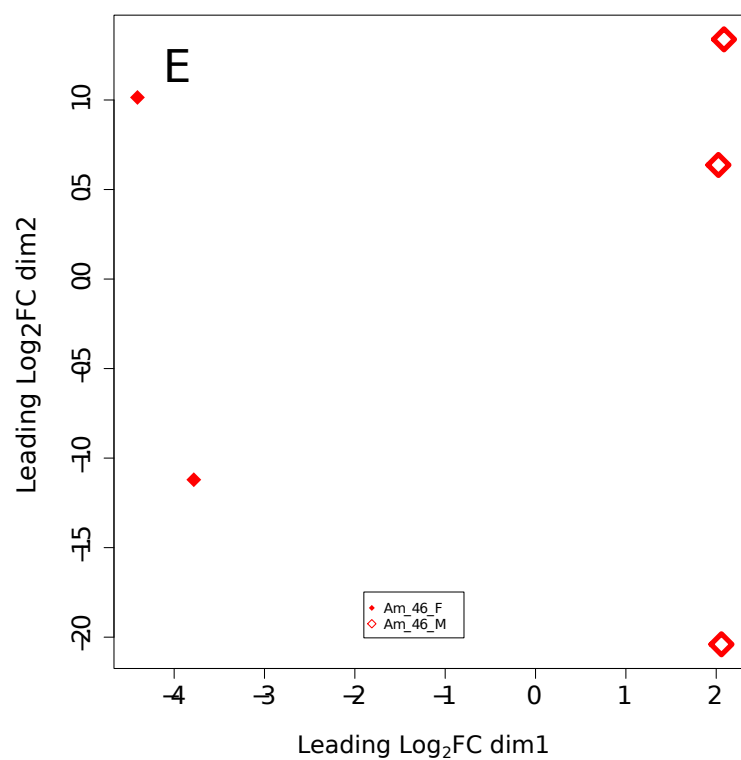

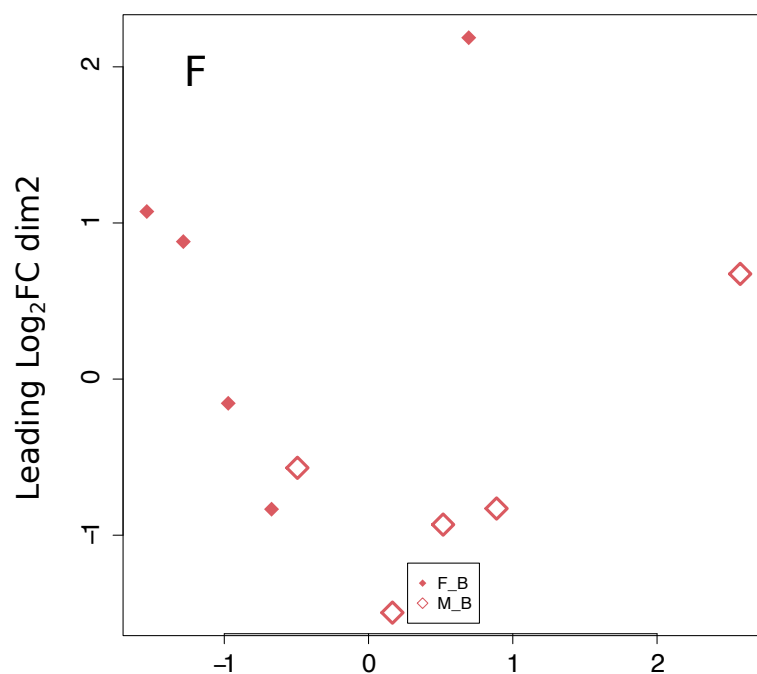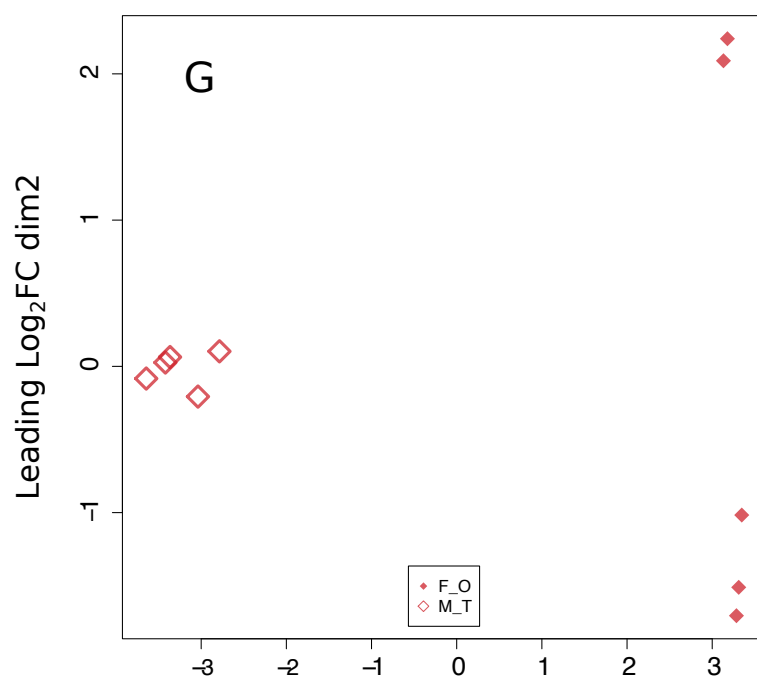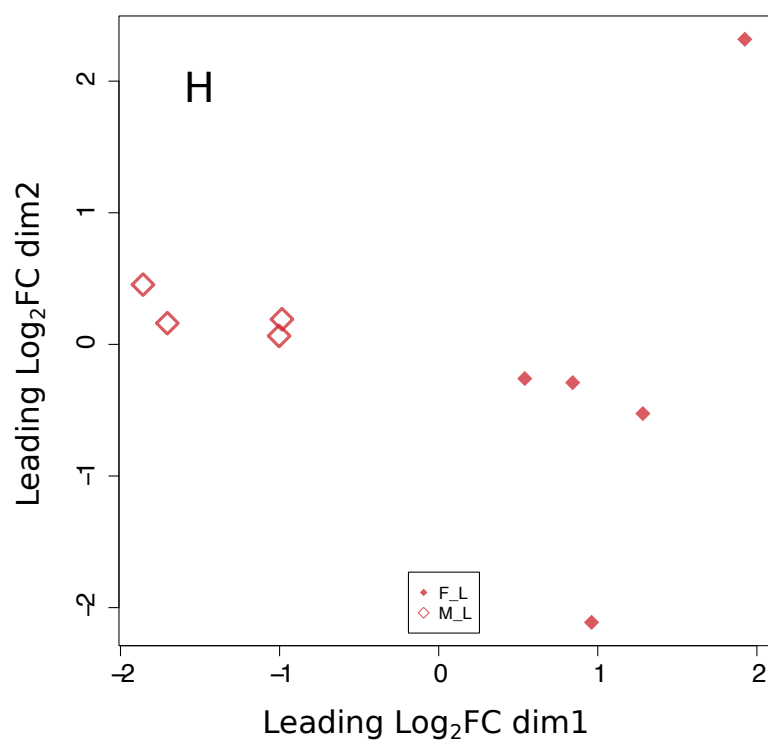

Supplement: Supplementary file 1 [file genes-09-00294-s001.zip › all_suppl/suppl_figures/FigureS1.pdf]

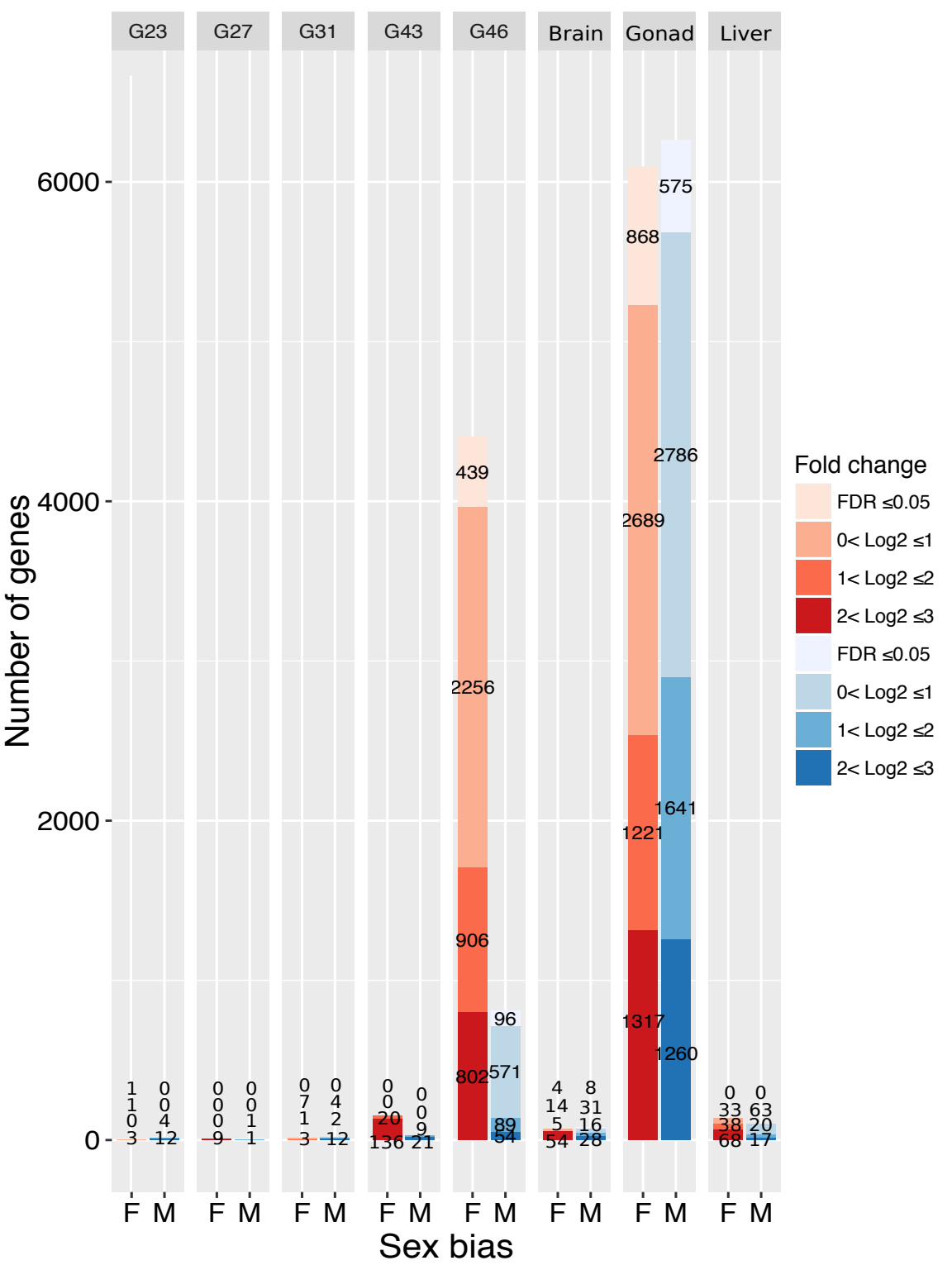

Supplement: Supplementary file 1 [file genes-09-00294-s001.zip › all_suppl/suppl_figures/FigureS4.pdf]

# A female-biased genes

G23

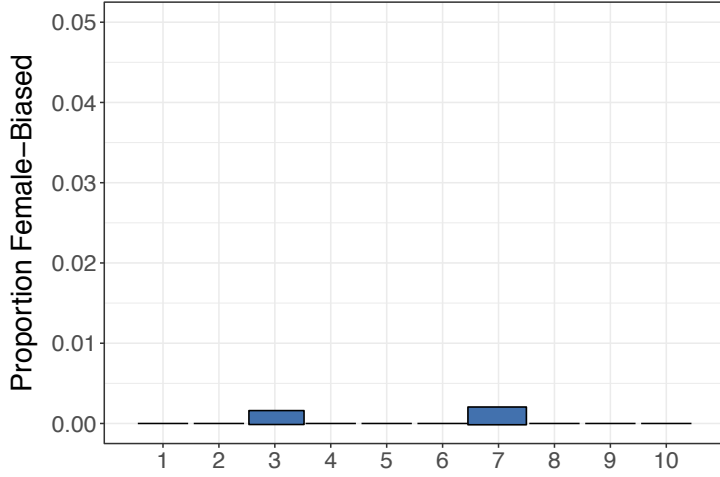

G27

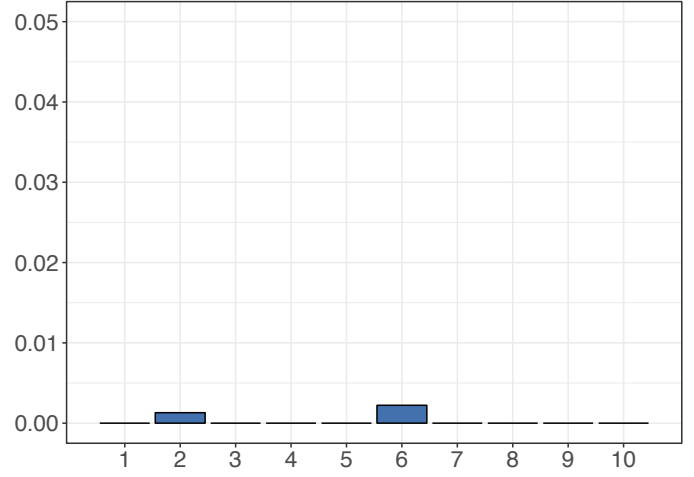

G31

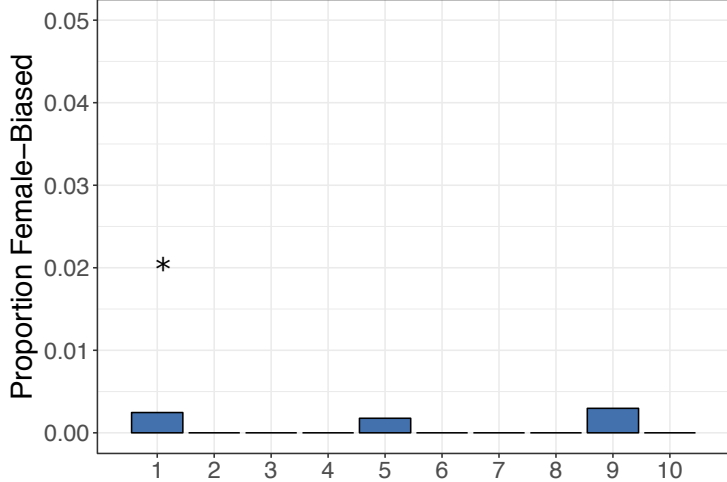

G43

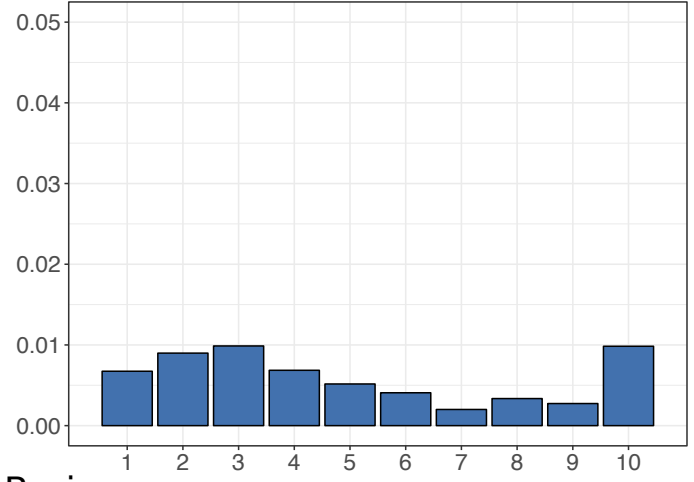

G46

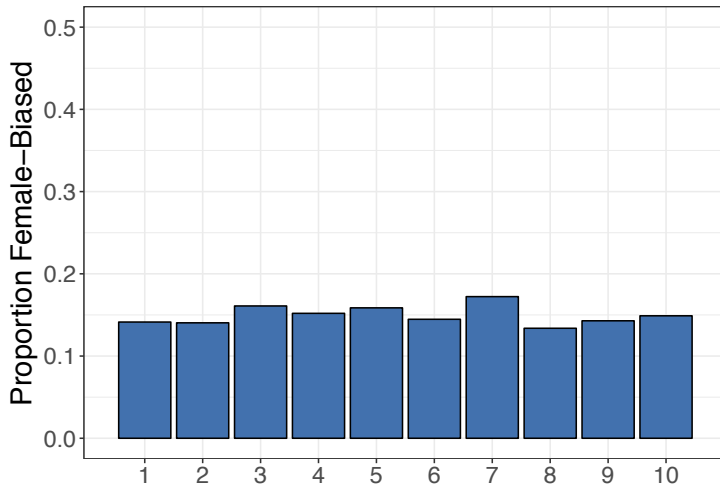

Brain

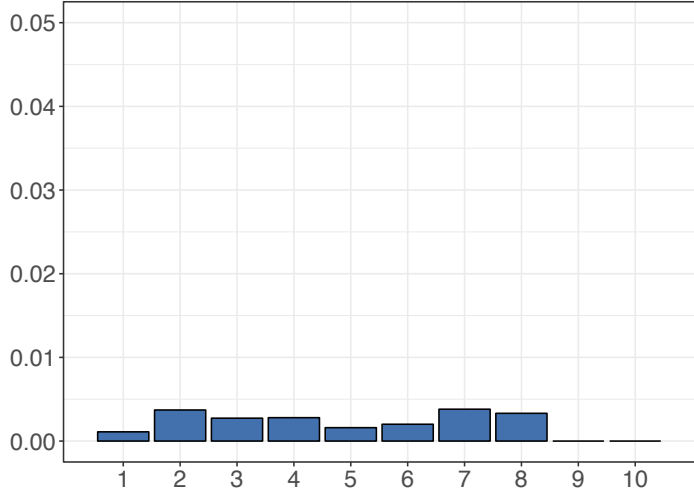

Gonad

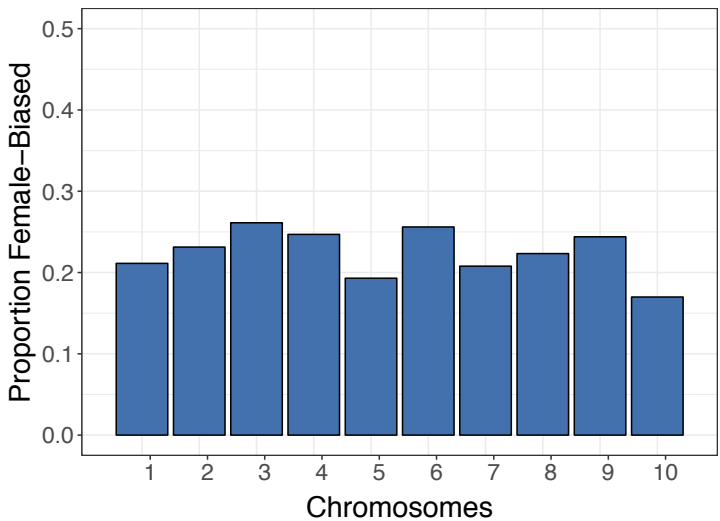

Liver

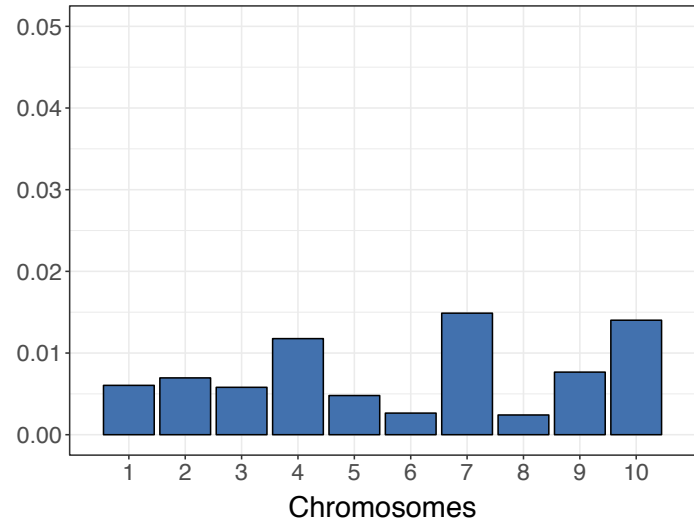

## B male-biased genes

# G23

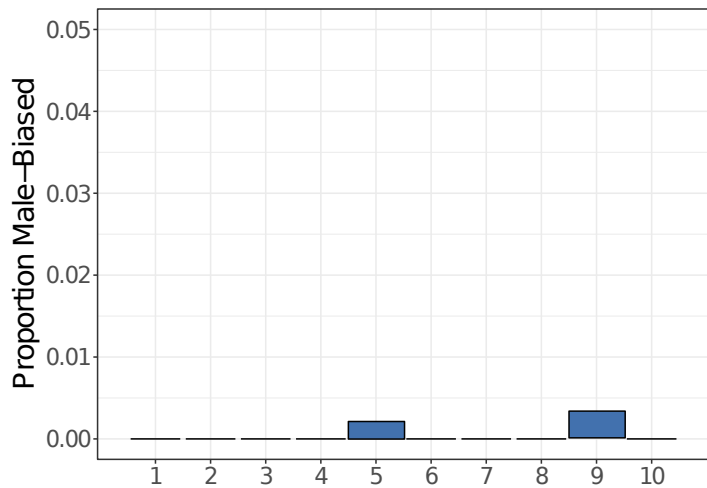

G27

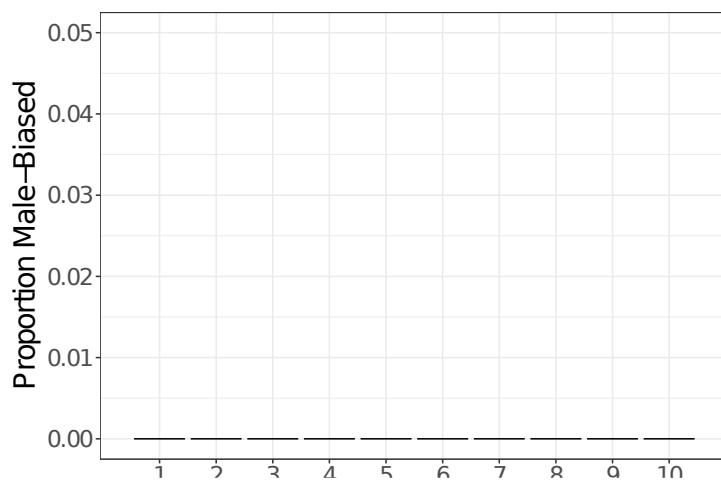

G31

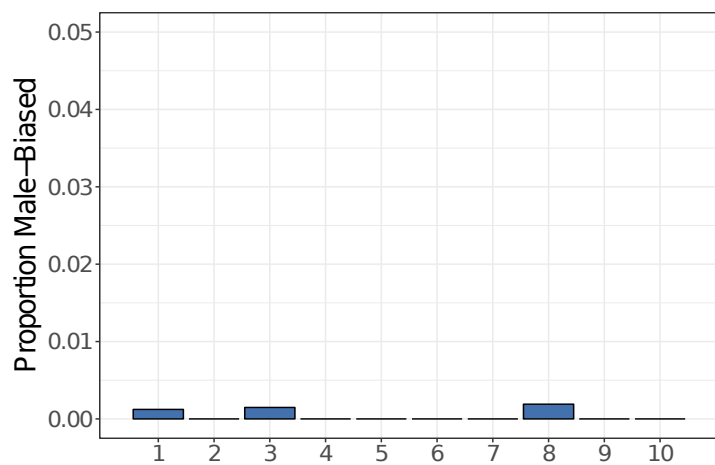

G43

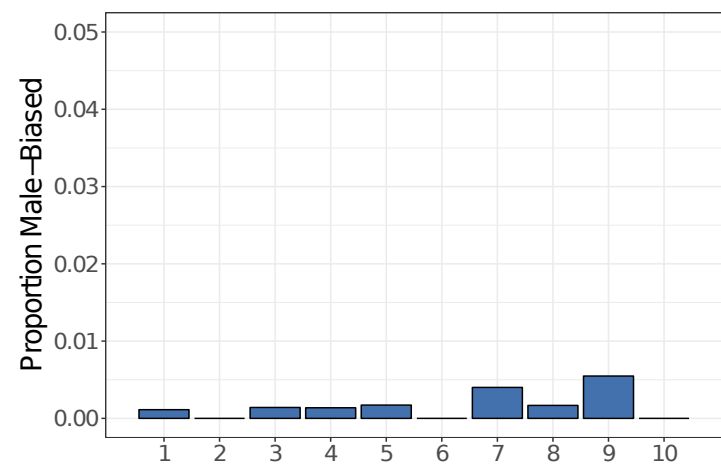

G46

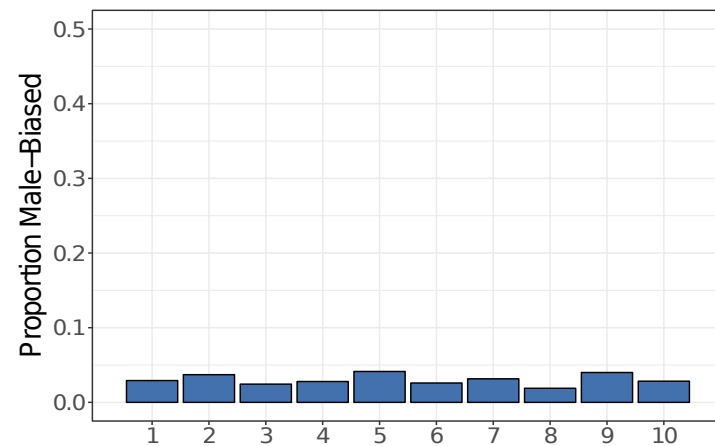

# Brain

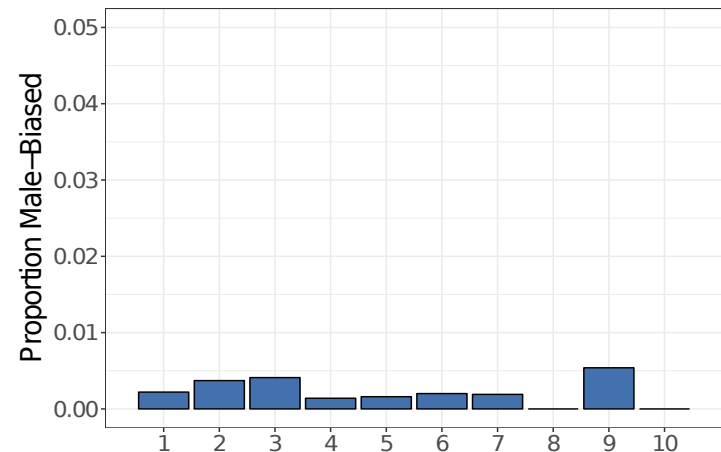

# Gonad

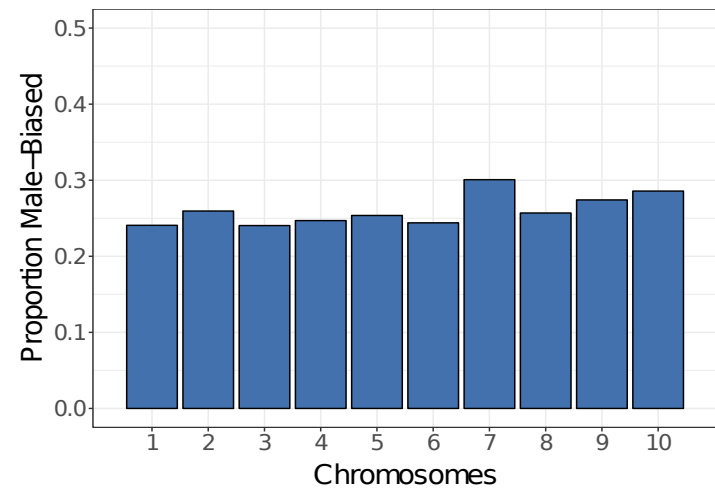

Liver

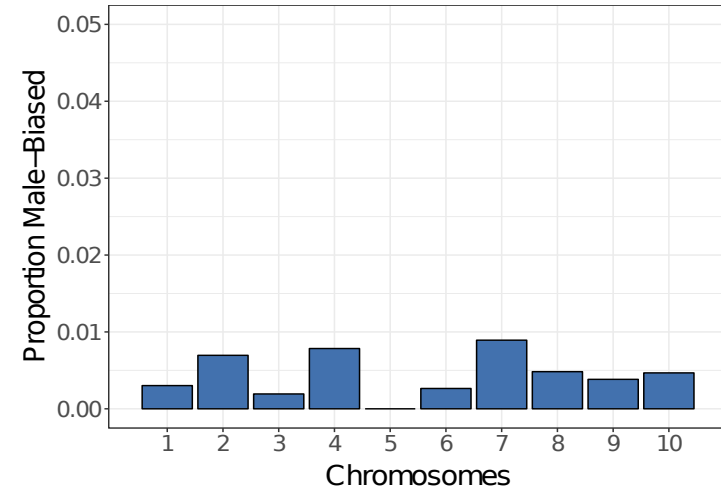

Supplement: Supplementary file 1 [file genes-09-00294-s001.zip › all_suppl/suppl_figures/FigureS5.pdf]

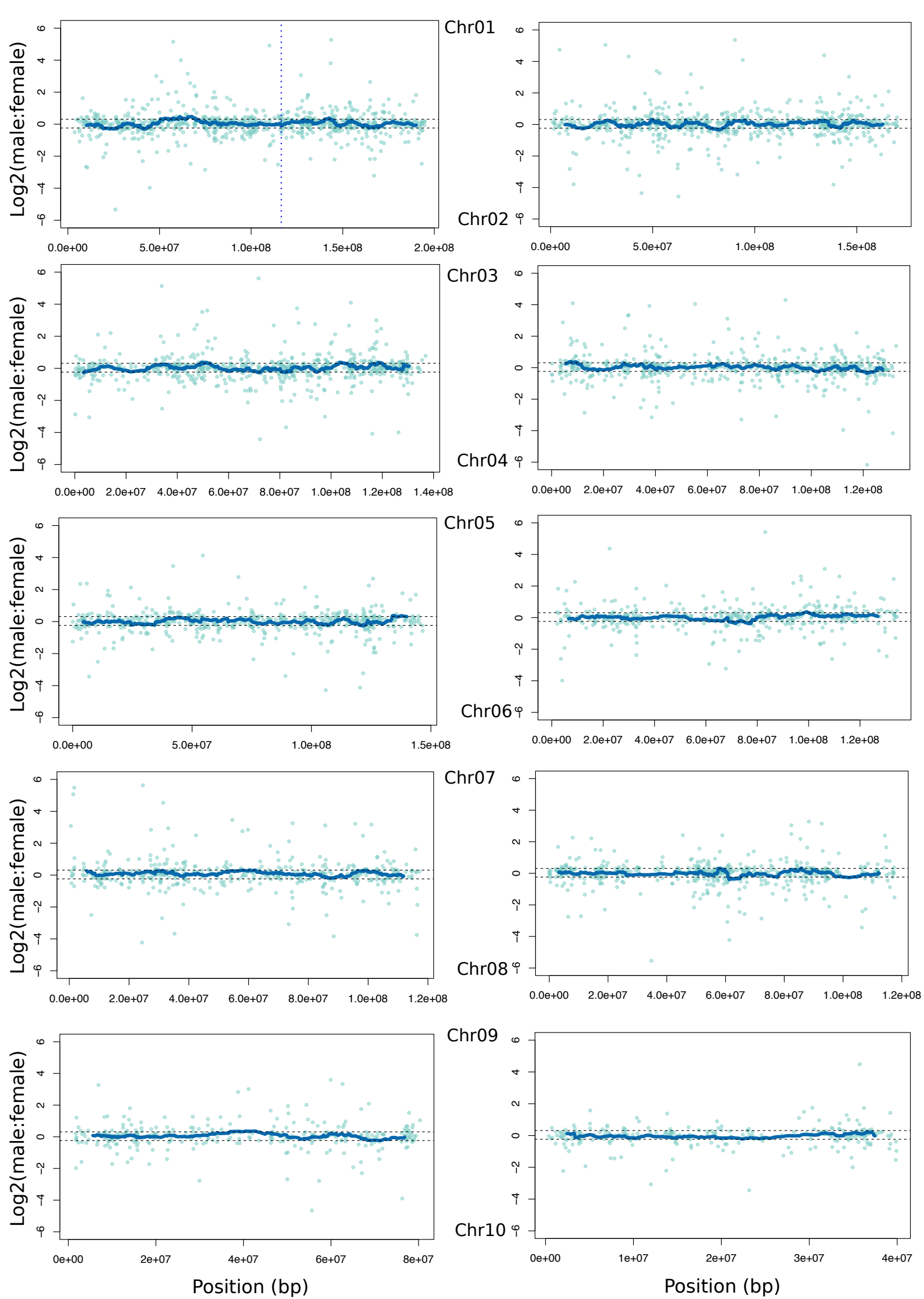

Supplement: Supplementary file 1 [file genes-09-00294-s001.zip › all_suppl/suppl_figures/FigureS7.pdf]

**G23**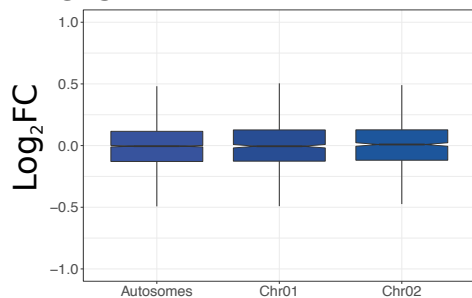**G27**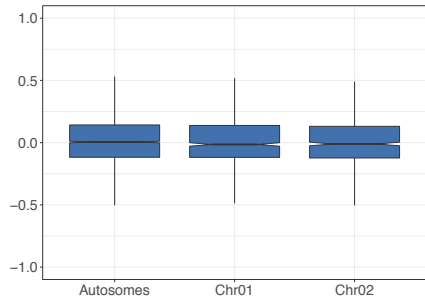**G31**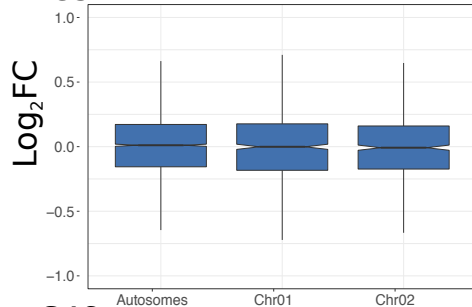**G43**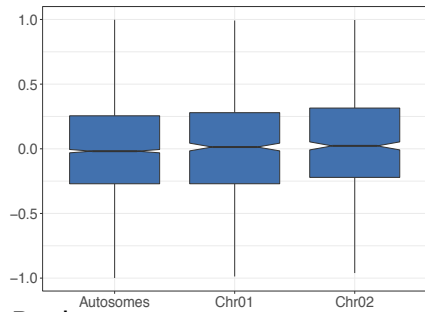**G46**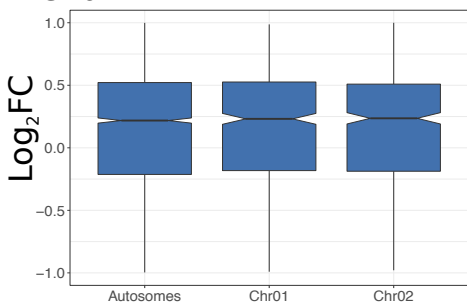**Brain**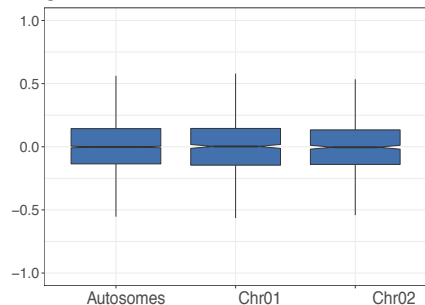**Gonad**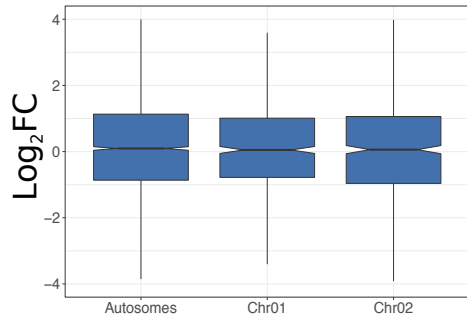**Liver**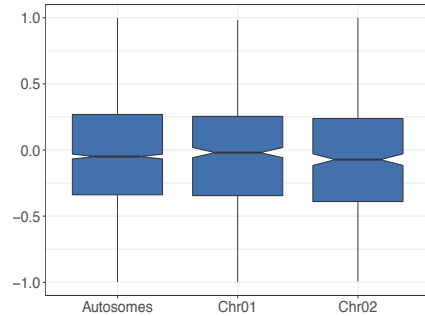

Supplement: Supplementary file 1 [file genes-09-00294-s001.zip › all_suppl/suppl_figures/FigureS6.pdf]
