# Supplementary material for: Tissue Specificity and Dynamics of Sex-Biased Gene Expression in a Common Frog Population with Differentiated, Yet Homomorphic, Sex Chromosomes
Source: Genes (Basel). 2018 Jun 12;9(6):294. doi: 10.3390/genes9060294 (PMC6027210; doi:10.3390/genes9060294)
Supplement: Supplementary file 1 [file genes-09-00294-s001.zip › all_suppl/Supplementary Materials.doc]

**Supplementary Materials caption**

**Figure S1**: Multidimensional scaling plot of gene expression data among samples within each tissue, (A) G23, (B) G27, (C) G31, (D) G43, (E) G46, (F) gonad, (G) brain, (H) liver.

**Figure S2**: Principle component analysis of gene expression data among all eight different tissues. (A) PC1 vs. PC2, (B) PC3 vs. PC4, (C) PC5 vs. PC6.

**Figure S3**: Venn diagram of sex-biased genes among (A) five developmental stages, (B) three adult tissues.

**Figure S4**: Summary of sex-biased genes defined by different fold-change threshold across five developmental stage tissues (G23, G27, G31, G43, G46), and three adult tissues (brain, gonad, liver).

**Figure S5**: Permutation test for chromosome enrichment compared to autosomal genes for (A) female-biased genes, (B) male-biased genes. Sex-biased genes are defined separately in each developmental stage and adult tissue. Stars denote significance level (*: *p* < 0.05).

**Figure S6**: Wilcoxon tests for Log_2_FC gene expression ratio differences between sex-linked and autosomal genes for five developmental stages and three adult tissues. Stars denote significance level (*: *p* < 0.05).

**Figure S7**: In G46 tissue, gene expression ratio Log_2_(male:female) over the length of sex chromosomes (Chromosome 1, and Chromosome 2), as well as each of 8 autosomes with sliding window of 40 genes. Dashed lines indicate the 95% confidence intervals, based on the Log_2_(male:female) ratios of all autosomal genes.

**Figure S8**: In gonad tissue, gene expression ratio Log_2_(male:female) over the length of sex chromosomes (Chromosome 1, and Chromosome 2), as well as each of 8 autosomes with sliding window of 40 genes. Dashed lines indicate the 95% confidence intervals, based on the Log_2_(male:female) ratios of all autosomal genes.

**Figure S9**: *dN/dS* ratios (a,b) and tissue specificity (c,d) of genes with turnover in sex-bias direction, and sex-biased genes being consistent in sex-bias direction (female, male respectively) between gonad and G46 tissues (a,c), and between gonad and liver tissues (b,d). Stars denote significance level (.: *p* < 0.1, *: *p* < 0.05, **: *p* < 0.01, *** *p* < 0.001).

**Figure S10**: Wilcoxon tests for differences in synonymous mutations, non-synonymous mutations and their ratio between the autosomes and sex chromosomes (A–C). Stars denote significance level (*: *p* < 0.05).

**Figure S11**: *dN/dS* ratios over the length of sex chromosomes (Chromosome 1, and Chromosome 2), as well as each of 8 autosomes with sliding window of 40 genes. Dashed lines indicate the 95% confidence intervals, based on the *dN/dS* ratios of all autosomal genes.

**Figure S12**: Permutation test for chromosome enrichment compared to autosomal genes for female (A,C,E), male-biased genes (B,D,F), as well as total Log_2_(male:female) gene expression ratio in stage G46 tissue, generated under different filtering criteria. (A,G) (total Log_2_CPM > 2), (C,H) (average Log_2_CPM > 0) are under relaxed filtering criteria, while (E) is from stringent filtering criteria (average Log_2_CPM > 0, and CPM > 1 in at least half of the samples per sex). Stars denote significance level (*: *p* < 0.05, **: *p* < 0.01, *** *p* < 0.001).

**Figure S13**: Permutation test for chromosome enrichment compared to autosomal genes for female (A,C,E), male-biased genes (B,D,F), as well as total Log_2_(male:female) gene expression ratio in gonad tissues, generated under different filtering criteria. (A,G) (total Log_2_CPM > 2), (C,H) (average Log_2_CPM > 0) are under relaxed filtering criteria, while € is from stringent filtering criteria (average Log_2_CPM > 0, and CPM > 1 in at least half of the samples per sex). Stars denote significance level (*: *p* < 0.05, **: *p* < 0.01, *** *p* < 0.001).

**Table S1**: *Dmrt1* genotyping of all samples used in RNAseq analysis and the indicated genotypic sex, phenotypic sex obtained from dissection under microscopy following scoring from *Ma* et al., 2016.

**Table S2**: Genomic distributions of one-to-one orthologs of reciprocal best blast hit between *Rana temporaria* and *Xenopus tropicalis*. * ratio between *R. temporaria* ortholog number and the number of *X. tropicalis* gene number per chromosome. *p* values of Chi-Square test is performed to compare orthologs on sex chromosome (Chr01) and each autosome. NS, non-significant, *p* > 0.05.

**Table S3**: Summary of super exact tests for overlap of sex biased genes between any two of the five developmental stages.

**Table S4**: Wilcoxon non-parametric test between sex-biased and unbiased gene expression in both female and male tissues, as well as comparison among sex-biased gene expression of various Log_2_FC cut off for G43, G46, gonad, brain and liver tissues. *p* < 0.05 is highlighted in bold.

**Table S5**: GO terms of sex-biased genes for G46 and three adult tissues.

**Table S6**: Wilcoxon rank sum test for rate of evolution (*dN/dS*) among female-biased, male-biased, and unbiased genes in tissues with >100 male biased genes. *p* < 0.05 are in bold.

**Table S7**: Spearman’s rank correlation test between Log_2_(male/female) gene expression ratio and tissue specificity Τ in tissues with <100 sex biased genes, such as G23, G27, G31 tissues.

**Table S8**: Wilcoxon rank sum test for tissue specificity Τ among female-biased, male-biased, and unbiased genes in tissues with >100 sex biased genes. *p* < 0.05 are in bold.

**Table S9**: Summary of linear models testing the effects of sex bias, tissue specificity and their interaction in the rate of coding sequence evolution. *p* < 0.05 are in bold.

**Text S1**: Method description of producing a *de novo* transposable element library from *Rana temporaria*.
