# Supplementary material for: Tissue Specificity and Dynamics of Sex-Biased Gene Expression in a Common Frog Population with Differentiated, Yet Homomorphic, Sex Chromosomes
Source: Genes (Basel). 2018 Jun 12;9(6):294. doi: 10.3390/genes9060294 (PMC6027210; doi:10.3390/genes9060294)
Supplement: Supplementary file 1 [file genes-09-00294-s001.zip › all_suppl/suppl_tables/TableS2.doc]

**Table S2**. Genomic location distributions of one-to-one ortholog of reciprocal best blast hit between *R. temporaria* and *X. tropicalis*. * ratio between *R. temporaria* ortholog number and the number of *X. tropicalis* gene number per chromosome. p values of Chi square test is performed to compare orthologs on sex chromosome (Chr01) and each autosome. NS, non-significant, p > 0.05.

| ***X. tropicalis* Chr.** | **Gene number** | ***R. temporaria* ortholog number** | **Ratio*** | **Chisq test** |
| --- | --- | --- | --- | --- |
| **1** | 3196 | 1555 | 0.49 | NS |
| **2** | 2646 | 1368 | 0.52 | NS |
| **3** | 2639 | 1216 | 0.46 | NS |
| **4** | 2325 | 1217 | 0.52 | NS |
| **5** | 2074 | 1029 | 0.50 | NS |
| **6** | 1645 | 839 | 0.51 | NS |
| **7** | 1940 | 838 | 0.43 | **0.02** |
| **8** | 2436 | 1034 | 0.42 | **0.005** |
| **9** | 1785 | 632 | 0.35 | **< 0.0001** |
| **10** | 948 | 514 | 0.54 | NS |
